# Statins regulate kinase signaling by causing changes in phosphorylation, rather than through changes in gene expression or direct inhibition: evidence in colorectal cancer

**DOI:** 10.3389/fphar.2025.1653702

**Published:** 2025-08-04

**Authors:** Francisco Alejandro Lagunas-Rangel, Jörgen Jonsson, Ludmila Jackevica, Robert Fredriksson, Maija Dambrova, Helgi B. Schiöth

**Affiliations:** ^1^ Laboratory of Pharmaceutical Pharmacology, Latvian Institute of Organic Synthesis, Riga, Latvia; ^2^ Department of Surgical Sciences, Functional Pharmacology and Neuroscience, Uppsala University, Uppsala, Sweden; ^3^ Department of Pharmaceutical Biosciences, Uppsala University, Uppsala, Sweden; ^4^ Faculty of Pharmacy, Riga Stradins University, Riga, Latvia

**Keywords:** cholesterol, HMGCR, cell signaling, kinome profiling, mevalonate

## Abstract

**Introduction:**

Statins, widely used for hypercholesterolemia, have shown anticancer properties including induction of apoptosis and ferroptosis, modulation of autophagy, and reprogramming of the tumor microenvironment, making them potential candidates for repurposing in cancer therapy. Although growing evidence suggests that statins may influence kinase signaling, current data remain inconclusive. To better understand this potential mechanism, we investigated the impact of statins on kinase activity.

**Methods:**

We employed an integrative approach combining publicly available RNA-seq and phosphoproteomic datasets with in vitro kinome inhibition profiling. The study assessed the effects of atorvastatin, simvastatin, and cerivastatin across a panel of 400 kinases. Western blot was used to assess whether reduced PI3K phosphorylation was due to mevalonate depletion.

**Results:**

Our analyses revealed that statins primarily influence kinase signaling via alterations in phosphorylation rather than through transcriptional regulation or direct inhibition. Phosphoproteomic data showed a general reduction in kinase phosphorylation, although some kinases exhibited increased activity. Affected kinases were significantly enriched in cancer-associated pathways, including insulin signaling, EGF–EGFR signaling, PI3K/AKT signaling, and the PD-L1/PD-1 immune checkpoint axis. Direct inhibition was observed for two kinases: CAMK1G (IC_50_ = 8.9 μM) and TSSK1B (IC_50_ = 3.3 μM). In colorectal cancer cell lines, decreased PI3K phosphorylation was at least partially attributable to mevalonate depletion, a known consequence of statin treatment.

**Discussion:**

These findings suggest that the anticancer activity of statins may be mediated, at least in part, through their ability to modulate kinase phosphorylation and activity. This mechanistic insight supports further exploration of statins as modulators of kinase signaling in oncology.

## 1 Introduction

Statins are inhibitors of 3-hydroxy-3-methylglutaryl-CoA reductase (HMGCR), the rate-limiting enzyme in the biosynthesis of cholesterol and non-sterol isoprenoids (Sirtori, 2014). This mechanism makes statins highly effective cholesterol-lowering agents and widely prescribed for the prevention and treatment of cardiovascular diseases (Mach et al., 2020). In recent years, growing interest has emerged in repurposing statins for cancer therapy (Lagunas-Rangel et al., 2024). Statins have demonstrated anticancer properties, including the induction of apoptosis and ferroptosis, modulation of autophagy, and reprogramming of the tumor microenvironment toward an anti-tumor state (Jiang et al., 2021; Lagunas-Rangel et al., 2025a; Lagunas-Rangel et al., 2025b). These findings suggest that statins may enhance the efficacy of conventional anticancer treatments and help overcome limitations such as drug resistance and limited treatment response (Matusewicz et al., 2020; Jiang et al., 2021).

Colorectal cancer is the third most common malignancy worldwide and the second leading cause of cancer-related mortality (Bray et al., 2024). Although advances in treatment have improved survival rates, significant challenges such as treatment resistance and non-response remain (Xie et al., 2020). These issues highlight the need to explore alternative or complementary therapeutic strategies. Cholesterol plays a relevant role in cell proliferation and progression through the cell cycle, particularly during the transition to the S phase (Singh et al., 2013). It is also a key component in the formation of lipid rafts in the cell membrane and is essential for vesicular trafficking, both of which are important for cell signaling and membrane dynamics (Silvius, 2003). In cancer cells, cholesterol demand increases significantly due to their rapid proliferation and enhanced mitogenic signaling (Xiao et al., 2023). To meet these demands, cancer cells often exhibit upregulation of the mevalonate pathway, which not only supports cholesterol synthesis, but also provides critical intermediates necessary to maintain several hallmarks of cancer, such as sustained growth, survival signaling, and membrane biosynthesis (Juarez and Fruman, 2021).

Dysregulated kinase activity, particularly overactivation, is a common hallmark of many cancers and plays a central role in promoting tumor growth, survival, and metastasis (Du and Lovly, 2018). Interestingly, there is increasing evidence that statins may affect kinases as part of a complex set of actions not directed at other proteins beyond HMGCR, although these mechanisms are not yet well understood (Lagunas-Rangel et al., 2024). In this context, a bioinformatics study previously suggested that simvastatin may interact with a broad range of kinases. Follow-up *in vitro* assays further reported direct inhibitory effects of simvastatin on EGFR (IC50 = 63.1 ± 8.2 nM), MET (IC50 = 22.9 ± 4.0 nM), and SRC (IC50 = 288.4 ± 35.7 nM) (Li et al., 2020). These findings raise the possibility that the anticancer properties of statins could also be mediated, at least in part, through their capacity to modulate kinase activity.

With this in mind, our study aimed to investigate how statins affect kinase regulation in colorectal cancer cells by examining their impact on gene expression, phosphorylation status and global kinase activity. We also aimed to determine whether these changes were associated with specific signaling pathways or biological processes. In particular, we explored whether the observed alterations in PI3K phosphorylation were a direct consequence of inhibition of the mevalonate pathway. Overall, the findings of this study provide new insights into the putative anticancer mechanisms of statins and may help to identify chemotherapeutic agents that could synergize with statins to improve therapeutic outcomes.

## 2 Materials and methods

### 2.1 RNA-seq data analysis

Publicly available RNA sequencing data were obtained from the Gene Expression Omnibus (GEO) under accession number GSE157167 (Norkin et al., 2021). This dataset includes transcriptomic profiles of colorectal cancer organoids derived from AKP mutant mice (harboring mutations in APC, KRAS and TP53) treated with vehicle (control), atorvastatin 1 µM or lovastatin 0.5 µM for 48 h. Sequencing was performed using the Illumina NextSeq 500 platform. First, the quality of the raw sequencing reads was assessed using the FastQC toolkit (Wingett and Andrews, 2018). Low quality bases and adapter sequences were removed with Trimmomatic (Bolger et al., 2014) to ensure data integrity. The cleaned reads were then aligned to the GRCm39 mouse reference genome (Release M37) using HISAT2 (Kim et al., 2019). Gene expression quantification was performed with featureCounts (Liao et al., 2014) and to focus the analysis on reliably expressed genes, low abundance transcripts, defined as those with a count per million (CPM) ≤1 in less than two samples per group, were filtered out. The normalized count data were then analyzed for differential expression using the edgeR package (Robinson et al., 2010). Genes were considered upregulated if they exhibited a log2 fold change (log2FC) >1.0 and a false discovery rate (FDR) <0.05. Conversely, genes with a log2FC <−1.0 and FDR <0.05 were classified as downregulated.

### 2.2 Phosphoproteomic data analysis

Phosphoproteomic data (publicly available) were obtained from the study by Ouahoud et al. (2021), which analyzed the phosphorylation landscape of colorectal cancer HCT116 cells treated with 2 µM lovastatin or vehicle control for 48 h. For the analysis, we specifically focused on phosphorylation changes in kinases following statin treatment. Kinase-associated phosphopeptides were identified, and their phosphorylation intensities were statistically analyzed to assess the significance between conditions. For each peptide, average data (Σ[x_1_, x_2_, …, x_9_]/n) and standard deviation (σ[x_1_, x_2_, …, x_9_]) between biological replicates within each treatment group were collected. To compare phosphorylation levels between the lovastatin-treated and control groups, Welch’s t-test for independent samples was applied. The degrees of freedom were estimated using the Welch–Satterthwaite approximation, and a *p*-value was computed for each phosphosite. To correct for multiple hypothesis testing, FDR adjustment was performed. Kinases were considered to exhibit significant changes in phosphorylation if FDR ≤0.05.

### 2.3 Functional enrichment analysis

Based on the phosphoproteomic data and the set of kinases that showed phosphorylation changes, we performed a pathway enrichment analysis to identify pathways and associated biological processes. This analysis was performed using the Kyoto Encyclopedia of Genes and Genomes (KEGG) database (Kanehisa, 2000) with default parameters.

### 2.4 Kinome screening

To investigate whether statins can directly inhibit specific kinases, we performed an initial screening using the SelectScreen™ Biochemical Profiling platform (Thermo Fisher Scientific), testing atorvastatin, cerivastatin, and simvastatin at a concentration of 1 µM. This screening covered 400 kinases, including both wild-type and cancer-associated mutants, using three fluorescence-based assay formats: LanthaScreen™, Adapta™, and Z′-LYTE™. The LanthaScreen™ assay detects kinase-mediated phosphorylation via a time-resolved fluorescence resonance energy transfer (TR-FRET) signal generated by a terbium-labeled antibody binding to a phosphorylated fluorescein-labeled substrate. The Adapta™ assay monitors ADP production by measuring the displacement of a labeled tracer from an antibody, with reduced TR-FRET signal indicating kinase activity and inhibitors preserving the signal by limiting ADP formation. The Z′-LYTE™ assay distinguishes phosphorylated from non-phosphorylated peptides based on their proteolytic susceptibility, with fluorescence changes reflecting kinase activity. Details on which assay was used for each kinase are provided in Supplementary Material S1. To validate the SelectScreen™ results with simvastatin, we used the scanMAX KINOMEscan^®^ platform (Eurofins Discovery), a high-throughput, site-directed competition binding assay that profiles over 500 kinase domain-containing human wild-type and mutant targets. Additionally, for a subset of kinases (ABL1, CAMK1G, CAMK2B, EGFR, ERBB2, ERK5, FLT3(ITD), MAP4K5, MET, PAK3, PIP5K1A, PIP5K2B, PRKCD, PRKD1, RIOK1, RIOK3, ROS1, SRC, SYK, TIE1, TSSK1B, and YANK3), binding affinities were further quantified using the KdELECT Binding LeadHunter Assay (Eurofins), which reports IC_50_ concentration values.

### 2.5 Chemicals

Atorvastatin (TCI, A2476), simvastatin (Sigma-Aldrich, S6196), and cerivastatin (Sigma-Aldrich, SML0005) were used in this study. Prior to each experiment, all statins were dissolved in pure ethanol to prepare a 100 mM stock solution, which was subsequently diluted in the appropriate cell culture medium to reach the desired working concentration. Mevalonolactone was obtained from BLDpharm (BD2924).

### 2.6 Cell lines and culture conditions

The cell lines used in this study were obtained from the American Type Culture Collection (ATCC). CaCo-2 cells (HTB-37) were maintained in Dulbecco’s Modified Eagle Medium (DMEM, Gibco, 10566016) supplemented with 20% fetal bovine serum (FBS, Sigma, F9665) and 1% penicillin-streptomycin solution (Sigma, P0781). HCT116 cells (CCL-247) were cultured in McCoy’s 5A Medium (Gibco, 16600082) supplemented with 10% FBS and 1% penicillin-streptomycin solution. All cells were incubated at 37°C in a humidified atmosphere containing 5% CO_2_.

### 2.7 Cell viability assays

Monocultures were established by seeding 10,000 cells in 100 µL of medium per well in 96-well plates (Thermo Fisher Scientific, 167008). After a 24-h pre-incubation period, cells were treated with atorvastatin or vehicle control (ethanol) according to a predefined plate layout.

The final ethanol concentration in the cell cultures was 0.1%. The plates were then incubated for 48 h under standard culture conditions (37°C, 95% humidity, 5% CO_2_). Following treatment, cell viability was assessed using the MTT assay (Sigma-Aldrich, 475989) according to the manufacturer’s instructions. A minimum of three independent biological replicates was performed for each experimental group.

### 2.8 Western blot

A total of 200,000 cells were seeded in 2 mL of culture medium per well in 6-well plates (Thermo Fisher, 145380). After a pre-incubation period of 24 h to allow cell adhesion, treatments were applied according to a predefined arrangement of the plates. Cells were exposed to statins alone, to statins combined with 200 µM mevalonolactone or to a control vehicle (ethanol). The final ethanol concentration in all cultures, including controls, was standardized to 0.1%. After treatment, cells were incubated for an additional 48 h before further analysis. Cells were harvested by scraping with a scalpel and lysed in RIPA buffer (Thermo Fisher, R0278) supplemented with a protease inhibitor cocktail (Roche, 11836170001) to extract total protein. Protein concentrations were determined using the Lowry method, and three biological replicates were prepared for each experimental condition. For each sample, 20 µg of total protein was loaded onto a mini-PROTEAN TGX gel (Bio-Rad, 4561094), separated by SDS-PAGE, and transferred onto a PVDF membrane (Invitrogen, IB24001) using the iBlot 2 system (Invitrogen). Membranes were blocked for 1 h at room temperature with 5% nonfat milk in PBS 1X, followed by overnight incubation at 4°C with primary antibodies diluted according to the manufacturer’s instructions. After three washes with PBST buffer (PBS +0.1% Tween-20), membranes were incubated for 1 h at room temperature with a goat anti-mouse secondary antibody (Invitrogen, 31430), followed by additional PBST washes. Membrane stripping was performed by incubating the blot in stripping buffer (1.5% glycine, 0.1% SDS, 1% Tween-20; pH 2.2) for 10 min at 37°C with constant agitation, followed by three washes with PBST buffer. After washing, the membrane was reblocked with 5% nonfat milk in PBS before reprobing. Actin (BD Biosciences, 612656) was used as a loading control and processed in parallel under the same conditions. The primary antibodies used were PI3K (Abbkine, Abp52199) and phosphorylated PI3K (Thr607) (Abbkine, Abp50495). Protein detection was performed using the SuperSignal West Pico PLUS chemiluminescent substrate (Thermo Fisher, 1863096), and bands were visualized with the Azure c400 imaging system (Azure Biosystems). Band intensities were quantified using Image Studio Lite software (LI-COR Biosciences). Expression levels were calculated as the phosphorylated PI3K/total PI3K ratio, and values were normalized to the control sample.

### 2.9 Statistical analysis

All statistical analyses and graph generation were performed using GraphPad Prism version 9 (GraphPad Software, La Jolla, CA, United States). The normality of the data was assessed using the Shapiro–Wilk test. For comparisons between multiple groups, one-way analysis of variance (ANOVA) followed by Tukey’s multiple comparisons test was applied. Differences were considered statistically significant at p ≤ 0.05.

## 3 Results

### 3.1 Statins do not broadly alter the transcription of kinases, but they do affect their phosphorylation state in colorectal cancer

To investigate the effects of statins on kinases, we performed analyses of RNA-seq data obtained from colorectal cancer organoids derived from AKP mice treated with atorvastatin or lovastatin. Focusing specifically on kinases, we found that only a small subset showed differential expression. Specifically, 8 kinases were differentially expressed in response to atorvastatin and 9 in response to lovastatin (Table 1). Atorvastatin resulted in upregulation of five kinases (PDK1, EGFR, ACVR1, EPHB3, MAP2K1) and downregulation of three (DCLK1, HCK, ERN2). In contrast, lovastatin downregulated all 9 differentially expressed kinases (MAP2K1, CDK6, TK1, EPHB2, AKT1, BMPR1A, EGFR, HCK, ACVR1). Interestingly, EGFR, ACVR1 and MAP2K1 were upregulated by atorvastatin but downregulated by lovastatin, highlighting that, despite sharing a common mechanism of action, these statins exert distinct transcriptional effects. HCK was the only kinase consistently downregulated by both statins.

**TABLE 1:** Kinases upregulated or downregulated following exposure to atorvastatin or lovastatin.

Statin	Kinase		logFC	FDR
Atorvastatin	Pyruvate dehydrogenase (acetyl-transferring)] kinase isozyme 1	Pdk1	11.99	2.53E-15
	Epidermal growth factor receptor	Egfr	3.89	5.71E-15
	Activin receptor type-1	Acvr1	3.63	1.07E-12
	Ephrin type-B receptor 3	Ephb3	2.28	5.95E-06
	Dual specificity mitogen-activated protein kinase kinase 1	Map2k1	2.23	5.61E-08
	Doublecortin domain-containing protein 3A	Dclk1	−7.98	0.0064
	Hematopoietic cell kinase	Hck	−4.37	0.0001
	Endoplasmic reticulum-to-nucleus signaling 2	Ern2	−4.33	0.0022
Lovastatin	Dual specificity mitogen-activated protein kinase kinase 1	Map2k1	−15.56	1.50E-05
	Cyclin-dependent kinase 6	Cdk6	−15.19	1.50E-05
	Thymidine kinase	Tk1	−14.32	1.50E-05
	Ephrin type-B receptor 2	Ephb2	−13.72	1.50E-05
	RAC-alpha serine/threonine-protein kinase	Akt1	−13.19	1.50E-05
	Bone morphogenetic protein receptor type-1A	Bmpr1a	−13.19	1.50E-05
	Epidermal growth factor receptor	Egfr	−10.09	0.0002
	Hematopoietic cell kinase	Hck	−9.32	0.0021
	Activin receptor type-1	Acvr1	−9.23	0.0027

Since kinase activity is mainly regulated through phosphorylation, we also analyzed phosphoproteomic data from HCT116 colorectal cancer cells treated with lovastatin or vehicle. Among the 90 kinases identified, 67 showed significant changes in phosphorylation (FDR ≤0.05). Among the 23 kinases that did not reach the FDR threshold of significance were PTK2B, EPHA4, TXK, FGR, VRK1, MYLK, ERBB3, SRC, MAP3K7, SGK3, CHUK, MAP3K11, MAP2K6, MAPK4, BTK, AXL, LIMK1, MAPK14 and PAK1. Consistently, 18 kinases showed increased phosphorylation at one or more sites following lovastatin treatment. These included FGFR1, BMX, EPHB1, TGFBR1, FGFR4, SGK2, MAP2K3, TBK1, PLK1, PRKAA2, DYRK1B, FES, NEK6, EIF2AK2, VAV2, SYK, PKN1, and PRKCQ. In contrast, 36 kinases exhibited significantly reduced phosphorylation levels at one or more sites after lovastatin exposure. This group included EGFR, PDPK1, RAF1, CSNK1E, CSNK2A1, AKT1, MAP2K1, ABL1, MTOR, LCK, ATM, IGF1R, PTK6, FLT3, LYN, MAPK12, STK6, GRK6, GSK3B, PHKG1, ALK, TEC, PAK2, TESK1, PRKAR2B, RPS6KA4, RPS6KA3, PDK1, PRKCA, PRKCZ, PRKCD, PRKD1, PRKDC, CDK16, CDK5, and CDK4. The remaining 13 kinases exhibited mixed phosphorylation responses to lovastatin treatment, with some phosphorylation sites showing increased phosphorylation and other sites showing decreased phosphorylation. These kinases included CHEK2, MKNK2, PDGFRB, ZAP70, AKT3, MET, MAP3K5, MAP3K11, PTK2, BRAF, CSK, TGFBR2, and KIT. Table 2 lists the kinases that showed significant phosphorylation along with detailed site information and corresponding FDR values.

**TABLE 2:** Kinases showing significantly altered phosphorylation levels after lovastatin treatment. Values highlighted in red indicate the condition (control or lovastatin-treated) in which phosphorylation was higher.

		Control		Lovastatin		
Kinase	Peptide	Σ(x1,x2, … ,x9)/n	σ(x1,x2,….,x9)	Σ(x1,x2, … ,x9)/n	σ(x1,x2,….,x9)	FDR
FGFR1	HHIDYYKKTTN	−4117.33	2581.51	4334.00	2919.05	0.0000
	FMAKVYSDPQP	5653.33	612.70	9359.67	3926.23	0.0342
BMX	VLDDQYVSSVG	776.33	1371.26	11557.33	622.47	0.0000
	TSLAQYDSNSK	11790.00	4230.70	19369.00	8094.10	0.0419
EPHB1	PGMKIYIDPFT	1756.67	1843.41	13281.67	1854.42	0.0000
TGFBR1	ISEGTTLKDLI	−2663.33	1470.68	7859.00	2429.12	0.0000
FGFR4	AVSEEYLDLRL	−2708.67	2112.95	−327.33	2401.90	0.0493
SGK2	EDTTSTFAGTP	−1882.33	733.74	4034.00	1538.71	0.0000
MAP2K3	GYLVDSVAKTM	−1506.00	1292.24	4794.33	1848.92	0.0000
TBK1	DEQFVSLYGTE	−2853.00	2790.34	9083.67	3190.20	0.0000
PLK1	GERKKTLAGTP	−1791.33	2096.84	1582.67	788.97	0.0026
PRKAA2	GEFLRTSAGSP	6878.00	1981.37	29322.33	1851.06	0.0000
DYRK1B	LGQRIYQYIQS	−2581.33	741.89	3602.33	781.38	0.0000
FES	EADGVYAASGG	4577.33	4817.08	20138.33	2374.58	0.0000
NEK6	TTAAHSLVGTP	−2452.67	2149.32	30528.67	4152.45	0.0000
EIF2AK2	TRSKGTLRYMS	−4641.67	1377.29	4687.67	1845.84	0.0000
VAV2	GGDDIYEDIIK	−5158.33	2425.10	−1411.00	635.91	0.0040
	NDDDVYRSLEE	−1878.33	2405.35	1411.33	1301.77	0.0069
PRKCQ	TTVELYSLAER	−6502.33	3141.71	14825.67	8550.51	0.0000
SYK	VSFNPYEPELA	21232.33	3713.19	44355.33	3604.36	0.0000
PKN1	GLYSRSGSLSG	−59.00	3213.11	19786.00	20527.07	0.0435
EGFR	SFLQRYSSDPT	7050.67	495.84	516.67	1698.17	0.0000
	AEEKEYHAEGG	9519.67	2294.93	3211.00	481.64	0.0000
	LPVPEYINQSV	559.33	1992.53	−1271.67	904.28	0.0414
	LVEPLTPSGEA	13076.00	1872.92	1498.00	575.23	0.0000
PDPK1	QARANSFVGTA	−4541.00	1683.44	−1078.33	1372.35	0.0004
	DDEDAYGNYDN	19198.67	1240.60	3054.67	2442.07	0.0000
RAF1	RGQRDSSYYWE	6502.67	1428.02	1486.00	1581.84	0.0000
	INRSASEPSLH	4106.33	2061.90	−1642.67	1406.29	0.0000
	RQRSTSTPNVH	10403.33	6064.09	4413.33	4446.04	0.0420
CSNK1E	GQLRGSATRAL	4076.00	1078.61	−2567.67	695.74	0.0000
	PPTGATANRLR	6563.00	1380.88	1517.67	954.09	0.0000
	LRGSATRALPP	26869.00	3601.69	2577.67	3313.11	0.0000
CSNK2A1	GLAEFYHPGQE	21032.00	8015.27	517.67	3416.95	0.0000
	SVPTPSPLGPL	4337.33	1352.34	901.67	2882.06	0.0136
AKT1	GATMKTFAGTP	548.33	1063.63	−2710.33	2332.78	0.0077
	LEDNDYGRAVD	26546.00	2475.40	5704.33	1347.94	0.0000
MAP2K1	GDAAETPPRPR	6047.00	1897.59	788.33	1185.46	0.0000
	PGRPLSSYGMD	2313.67	3431.50	−1337.00	1271.16	0.0197
	DSMANSFVGTR	4026.67	946.06	−698.00	966.92	0.0000
ABL1	NKPTVYGVSPN	14916.00	1899.15	1669.00	1516.39	0.0000
	PGIDLSQVYEL	6618.33	1693.34	2661.33	4422.24	0.0421
MTOR	PESIHSFIGDG	12932.67	1071.61	6205.67	3874.95	0.0019
	RTRTDSYSAGQ	2778.33	729.99	−3288.33	1099.00	0.0000
LCK	IEDNEYTAREG	8424.33	2907.27	3789.00	2910.49	0.0073
ATM	AFEEGSQSTTI	10771.67	3852.50	4968.33	2783.11	0.0060
IGF1R	MTRDIYETDYY	21039.67	3419.71	13517.67	1606.00	0.0003
PTK6	IKEDVYLSHDH	9374.33	561.74	4050.67	2157.80	0.0003
FLT3	DNEYFYVDFRE	26299.00	5400.91	3741.67	2789.02	0.0000
LYN	TATEGQYQQQP	4281.00	5152.44	−3213.67	3417.98	0.0075
MAPK12	SEMTGYVVTRW	6392.67	2136.08	3003.33	2685.18	0.0000
STK6	SSRRTTLAGTL	9984.33	8135.40	936.33	2203.45	0.0263
GRK6	IEQFSTVKGVE	5460.67	1798.82	−947.67	1778.09	0.0000
GSK3B	PVQQPSAFGSM	4362.33	2061.25	1974.33	898.98	0.0146
PHKG1	ILRKVSGHPNI	4302.67	2269.68	1547.33	1058.74	0.0153
ALK	PGAGHYEDTIL	3557.33	2558.02	−2096.67	1828.25	0.0003
TEC	ERGQEYLILEK	5267.00	1141.58	−2058.67	609.83	0.0000
TESK1	LAVVGSPYWMA	6646.67	2018.09	287.00	1815.84	0.0000
PRKAR2B	FTRRASVAAEA	31610.33	4980.27	2504.00	2698.57	0.0000
RPS6KA4	YSPPGSPPPGD	6922.00	1710.54	1217.67	651.19	0.0000
RPS6KA3	KTPKDSPGIPP	7198.67	4771.16	2172.33	1068.46	0.0326
PDK1	ALSTDSIERLP	5060.33	1470.45	−361.67	563.93	0.0000
PRKCA	FEGFSYVNPQF	36948.00	4410.98	−1209.67	1644.57	0.0000
	DFEGFSYVNPQ	4078.33	2251.62	−3019.67	4911.67	0.0048
PAK2	DVLKFYDSNTV	18993.67	3790.32	11757.67	5295.50	0.0117
	SIYTRSVIDPV	3058.00	2725.55	1253.00	2187.87	0.0168
PRKCZ	EPVQLTPDDED	6196.67	1501.85	−1736.67	1553.04	0.0000
PRKCD	FDAHIYEGRVI	16524.00	7499.56	−2726.33	631.25	0.0003
	EKARLSYSDKN	3774.33	1915.67	597.00	1249.35	0.0011
PRKD1	GWMVHYTSKDT	7531.00	1014.23	2817.33	2732.62	0.0018
	IIGEKSFRRSV	1061.00	1061.00	−561.33	4292.24	0.0000
PRKDC	TLQTRTQEGSL	5626.33	2727.10	756.67	264.91	0.0019
CDK16	IKRQLSMTLRG	8622.33	4659.42	301.17	2396.68	0.0000
CDK5	PVRAYSAEVVT	6804.67	997.00	−1419.67	1672.77	0.0000
CDK4	YQMALTPVVVT	13648.33	1329.63	5221.67	4788.45	0.0018
CHEK2	LETVSTQELYS	8975.33	2251.68	1253.33	717.16	0.0000
	VLAQPSTSRKR	−2978.00	2102.75	4080.00	2004.07	0.0000
PDGFRB	TSSVLYTAVQP	−2175.67	2853.90	5451.00	782.47	0.0000
	DESVDYVPMLD	14760.33	2684.79	7072.67	2923.32	0.0000
ZAP70	LVNRHYAKISD	13428.33	1769.22	29257.67	14555.28	0.0175
	ADDSYYTARSA	4419.67	1650.01	−1689.00	1648.92	0.0000
AKT3	AATMKTFAGTP	21286.00	7413.92	3111.00	643.82	0.0003
	ERMNASPTSQI	−544.33	1185.66	9996.33	7943.67	0.0073
MET	YDKEYYSVHNK	17670.67	3767.71	1209.00	4547.21	0.0000
MKNK2	ENTLPTPMVLQ	−5824.67	1201.75	4438.67	4276.39	0.0003
	FELAFSLDQPD	−277.67	833.39	−1980.67	865.18	0.0015
MAP3K5	NPATETFTGTL	35.67	2395.74	5860.00	1709.54	0.0000
	RGRGSSVGGGS	16219.33	875.61	1194.00	558.21	0.0000
MAP3K11	REWHKTTQMSA	3447.00	3136.17	9554.67	5639.17	0.0341
	KTTQMSAAGTY	12158.33	2403.02	−1391.00	2231.59	0.0000
PTK2	SNDKVYENVTG	12593.00	730.25	4989.33	2579.08	0.0000
	EDSTYYKASKG	−1448.67	598.60	2396.33	1439.08	0.0000
	SETDDYAEIID	7863.33	8317.17	−1417.67	2593.45	0.0164
BRAF	ERKSSSSSEDR	−9.33	1853.67	18297.67	7445.76	0.0003
	RDRSSSAPNVH	34991.00	5899.79	9290.67	13513.49	0.0007
CSK	AQDEFYRSGWA	−4156.00	963.52	−2540.00	853.76	0.0043
	REKKFSTKSDV	5943.67	3273.61	2071.00	1679.96	0.0144
TGFBR2	VGTARYMAPEV	15585.00	7034.63	2835.33	1163.08	0.0018
	DRSDISSTAAN	−476.67	2474.74	2855.33	757.18	0.0069
KIT	INGNNYVYIDP	19399.00	4079.36	6810.00	4561.72	0.0000
	DSTNEYMDMKP	−1236.00	1214.04	11457.67	2477.34	0.0000

**FIGURE 1:**
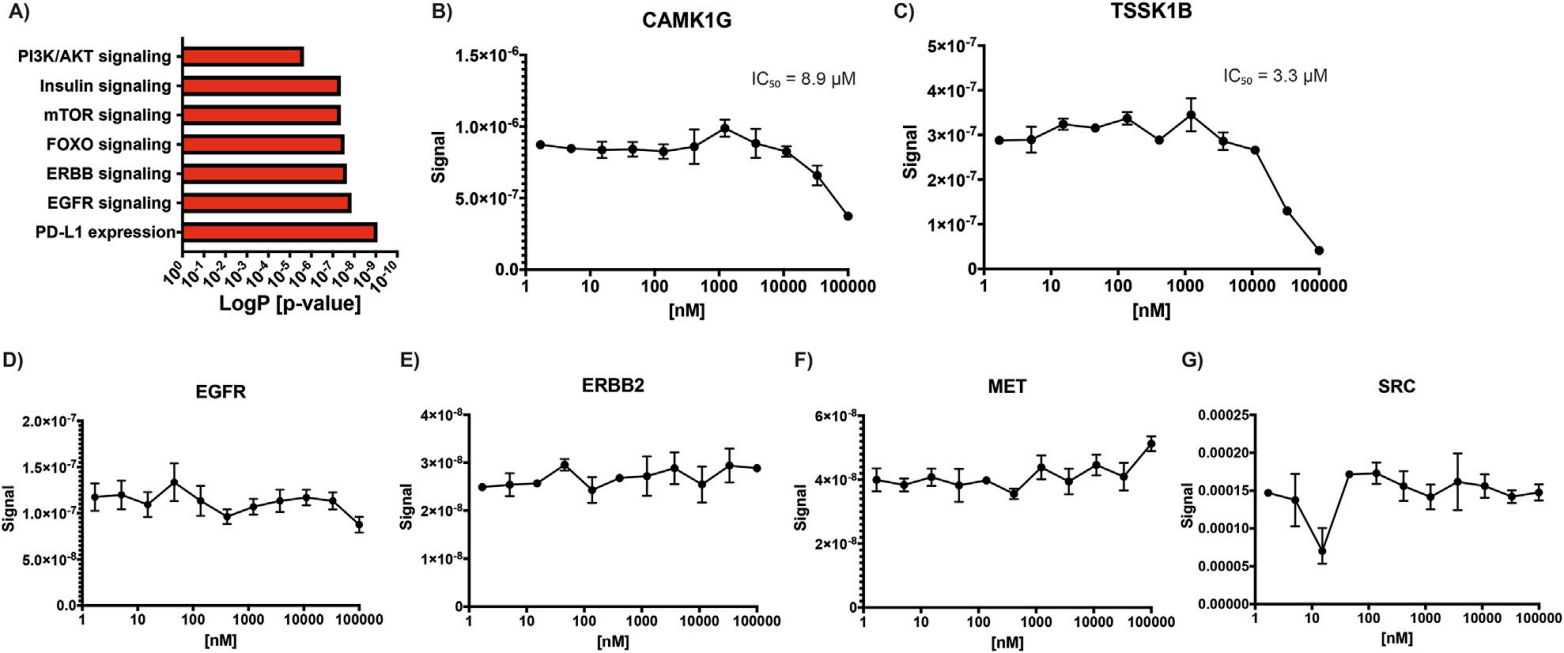
Statin effects on kinase activity. **(A)** KEGG pathway enrichment analysis of kinases showing decreased phosphorylation following lovastatin treatment compared to control, highlighting affected signaling pathways. **(B–G)** Simvastatin concentration-response curves used to calculate IC_50_ values for selected kinases: **(B)** CAMK1G, **(C)** TSSK1B, **(D)** EGFR, **(E)** ERBB2, **(F)** MET, and **(G)** SRC.

Functional enrichment analysis of kinases with decreased phosphorylation following lovastatin treatment revealed significant involvement in several key signaling pathways, including insulin signaling (*p* adjusted [adj] = 4.396 × 10^−8^), EGFR signaling (*p* adj = 1.395 × 10^−8^), ERBB signaling (*p* adj = 2.303 × 10^−8^), FOXO signaling (*p* adj = 2.940 × 10^−8^), mTOR signaling (*p* adj = 4.396 × 10^−8^), PI3K/AKT signaling (*p* adj = 2.296 × 10^−6^), and PD-L1 expression and the PD-1 checkpoint pathway in cancer (*p* adj = 8.690 × 10^−10^) (Figure 1A). In contrast, kinases with increased phosphorylation did not show enrichment in any specific biological process.

### 3.2 Statins rarely inhibit the activity of kinases by direct interaction

Given previous reports indicating that simvastatin can inhibit kinases such as EGFR, ERBB2, MET, and SRC, we sought to investigate whether other kinases might also be directly inhibited by statins (Li et al., 2020). To address this, we solicited a kinome profiling assay using atorvastatin, simvastatin, and cerivastatin across a panel of 400 kinases, including those previously reported to be statin-sensitive. A starting concentration of 1 µM was selected, as it was considered sufficiently high to detect potential inhibitory effects on susceptible kinases. However, the results from this initial screen were negative for all tested kinases, with none showing more than 20% inhibition, including EGFR, HER2, MET, and SRC (Supplementary Material S1). To confirm these findings, a second kinome profiling assay for simvastatin was performed by a different provider under identical conditions. This second screen included an expanded panel of 500 kinases and yielded similar results, confirming the lack of inhibition for most kinases. Notably, two kinases, CAMK1G and TSSK1B, showed greater than 95% inhibition by simvastatin in this second assay. To further investigate, concentration-response curves were generated to determine the IC_50_ values for these two kinases, along with a selected group of additional kinases (ABL1, CAMK1G, CAMK2B, EGFR, ERBB2, ERK5, FLT3(ITD), MAP4K5, MET, PAK3, PIP5K2B, PRKCD, PRKD1, RIOK1, ROS1, SRC, SYK, TIE1, TSSK1B, and YANK3). The IC_50_ for CAMK1G was determined to be 8.9 µM (Figure 1B), while that for TSSK1B was 3.3 µM (Figure 1C). Concentration curves were also generated for EGFR (Figure 1D), ERBB2 (Figure 1E), MET (Figure 1F), and SRC (Figure 1G) to reconcile our findings with previously published data. However, no inhibition was observed for these kinases at any tested concentration, and thus, IC_50_ values could not be determined, further supporting our conclusion that these kinases are not directly inhibited by simvastatin under the tested conditions.

### 3.3 Atorvastatin inhibits PI3K phosphorylation through mevalonate deficiency in colorectal cancer cells

**FIGURE 2:**
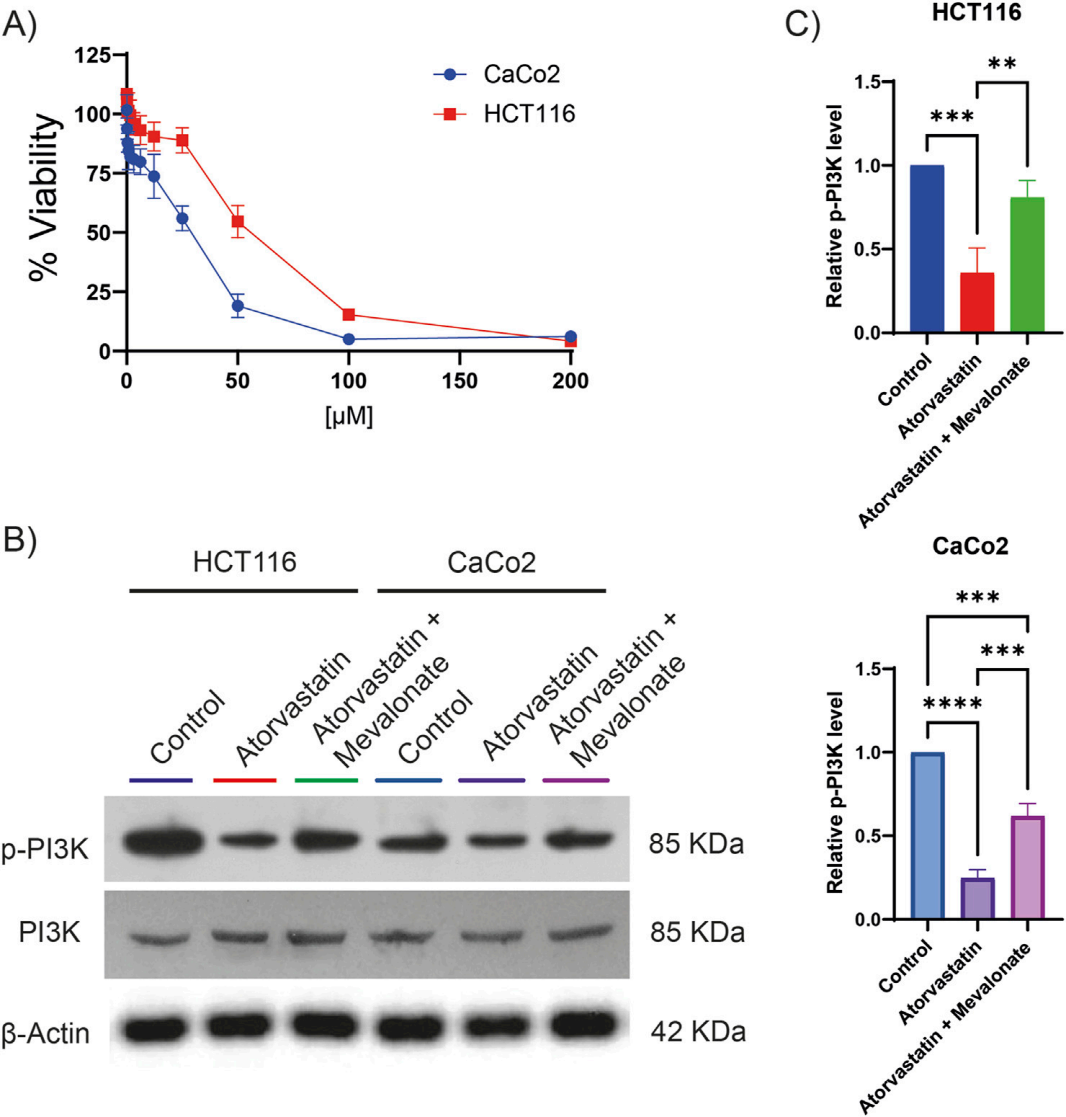
Atorvastatin inhibits PI3K phosphorylation in colorectal cancer cell lines. **(A)** Cell viability assays were performed to determine the IC_50_ of atorvastatin in HCT116 and CaCo2 colorectal cancer cells. The calculated IC_50_ values were 80.89 µM for HCT116 cells and 28.97 µM for CaCo2 cells. **(B)** Representative Western blot analysis showing total and phosphorylated PI3K levels in HCT116 and CaCo2 cells following treatment with atorvastatin. Three independent experiments were conducted. **(C)** Quantification of phosphorylated PI3K levels based on densitometric analysis of western blots. Phosphorylation was expressed as the ratio of phosphorylated PI3K to total PI3K, normalized to control levels. Statistical significance is indicated as **p ≤ 0.01, ***p ≤ 0.001, and ****p ≤ 0.0001.

Given that phosphoproteomic analysis revealed a decrease in PI3K phosphorylation following lovastatin treatment, we investigated whether a similar effect could be observed with atorvastatin, one of the most widely used statins globally and actively explored for cancer therapy. To begin, the citotoxic half-maximal inhibitory concentration (IC_50_) of atorvastatin was determined in two colorectal cancer cell lines, HCT116 and CaCo2 (Figure 2A). The calculated IC_50_ was 80.89 µM for HCT116 cells and 28.97 µM for CaCo2 cells. These concentrations were subsequently used for all downstream experiments. Atorvastatin treatment led to a significant reduction in PI3K phosphorylation in both cell lines (Figures 2B,C), confirming results previously observed with lovastatin. Since statins inhibit HMGCR, thereby blocking the conversion of HMG-CoA to mevalonate, we hypothesized that the observed reduction in PI3K phosphorylation could be attributed to mevalonate depletion. To test this, rescue experiments were performed by supplementing atorvastatin-treated cells with 200 µM mevalonolactone. This intervention restored PI3K phosphorylation to near-control levels in HCT116 cells and to approximately 60% in CaCo2 cells (Figures 2B,C). These results suggest that the decrease in PI3K phosphorylation is largely driven by mevalonate depletion resulting from atorvastatin-mediated HMGCR inhibition, although other mechanisms may be involved.

## 4 Discussion

**FIGURE 3:**
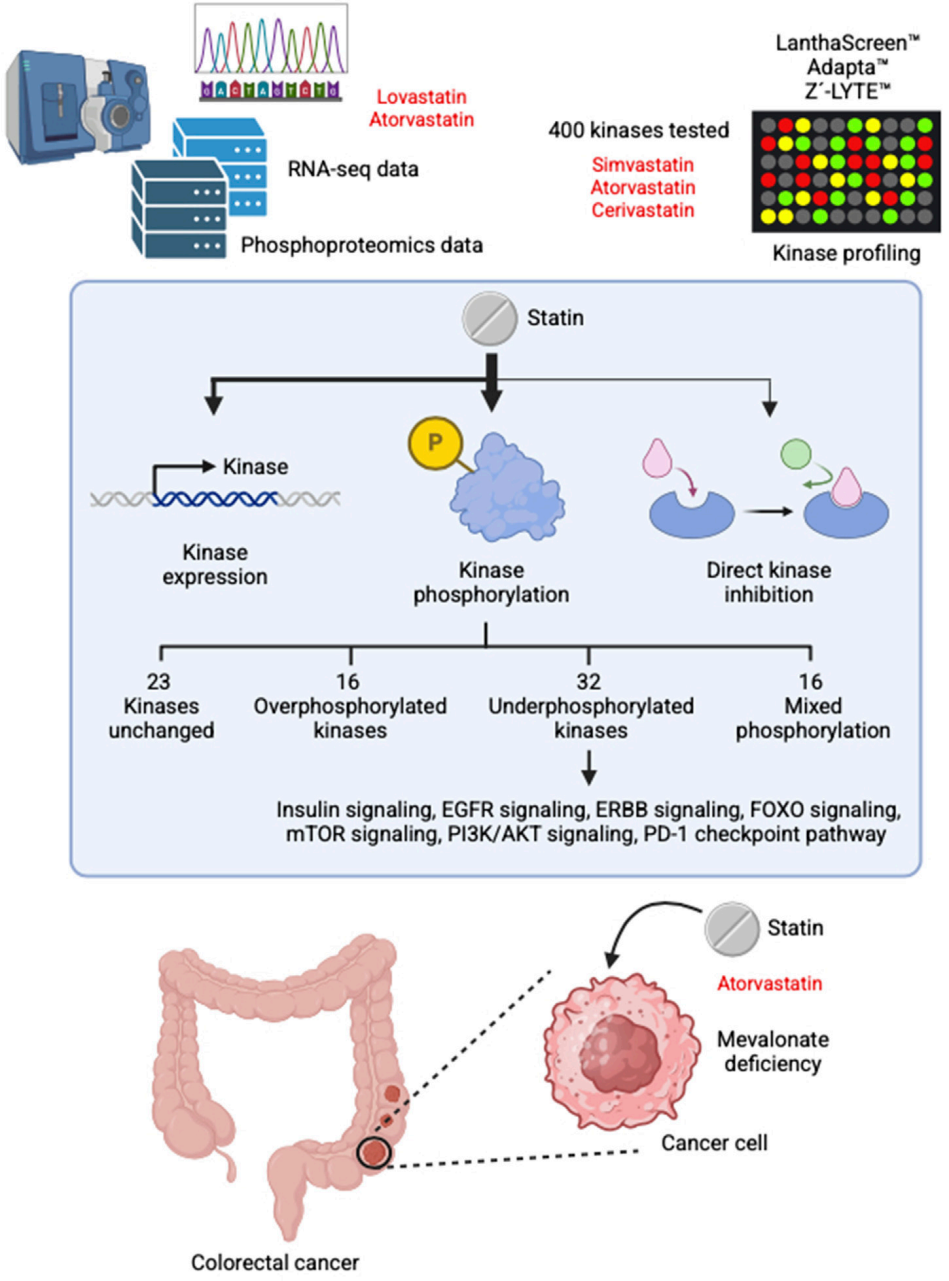
Summary of the main findings of this study.

This study shows that statins regulate kinase signaling primarily by causing changes in phosphorylation, rather than through changes in gene expression or direct kinase inhibition. This preference for phosphorylation-based regulation may be attributed to the fact that phosphorylation is a faster, more energy-efficient, and highly dynamic mechanism for controlling protein activity (Cozzone, 1998). In most cases, statin treatment led to a reduction in kinase phosphorylation, although increased phosphorylation was also observed for a subset of kinases. Notably, many of the affected kinases are involved in key signaling pathways associated with cancer development and progression, suggesting that the anticancer effects of statins are at least partially mediated through their effects on kinase activity. We also demonstrated that, in the case of PI3K, reduced phosphorylation is at least partly attributable to mevalonate depletion. However, this mechanism alone does not fully explain all phosphorylation changes observed, indicating that additional, as-yet-unidentified pathways may contribute. Figure 3 presents a concise overview of the principal results of this study.

Among the pathways most affected by statin-induced changes in kinase phosphorylation are insulin signaling, EGF–EGFR signaling, PI3K/AKT signaling, and the PD-L1/PD-1 immune checkpoint pathway in cancer. In this regard, previous studies have indicated that statins may contribute to insulin resistance through a variety of mechanisms, including impaired insulin signaling (Bredefeld et al., 2024), mitochondrial dysfunction (Mollazadeh et al., 2021), altered adipokine levels (Perelas et al., 2010), and modification of gut microbiota (She et al., 2024; Lagunas-Rangel et al., 2025a; Lagunas-Rangel et al., 2025b). Our findings add to this knowledge by suggesting that statin-induced modulation of kinase activity may also contribute. Notably, the EGF–EGFR and PI3K/AKT pathways are important for cell survival and proliferation (Liu et al., 2020; Levantini et al., 2022; Lagunas-Rangel et al., 2023), providing a mechanistic explanation for the antiproliferative effects of statins in cancer cells. EGFR and its downstream signaling pathways (including the PI3K/AKT pathway) play a key role in the development and progression of colorectal cancer. Upon activation, EGFR triggers a cascade of molecular events leading to the expression of genes involved in key cellular processes such as proliferation, migration, differentiation and apoptosis (Napolitano et al., 2024). Given its pivotal role in tumor biology, targeting the EGFR signaling axis at various molecular levels has become a key therapeutic strategy, especially in the treatment of metastatic colorectal cancer (Khan et al., 2019). Inhibition of the PD-L1/PD-1 checkpoint pathway is particularly relevant, as this pathway contributes to immune evasion and tumor progression (Ai et al., 2020). Although the improved tumor response to statin therapy has been largely attributed to the reduction of cholesterol levels and its metabolites in the tumor microenvironment (Lagunas-Rangel et al., 2025a; Lagunas-Rangel et al., 2025b), our data now suggest that modulation of kinase activity may also contribute to this immunomodulatory effect.

In cases where the kinase shows increased phosphorylation at some sites and decreased phosphorylation at others, the interpretation of the biological outcome becomes more complex. For example, following lovastatin exposure, CHEK2 exhibits reduced phosphorylation at T68 (LETVSTQELYS), a site phosphorylated by ATM in response to DNA damage and during the G2/M checkpoint (Ahn et al., 2000). Conversely, it increases phosphorylation at S456 (VLAQPSTSRKR), a site that promotes CHEK2 ubiquitination and degradation (Kass et al., 2009). This pattern suggests that statins may partially inhibit cancer DNA repair mechanisms by altering the normal phosphorylation dynamics of key kinases such as CHEK2 and ATM (which also decreases their phosphorylation), thereby weakening the cellular response to DNA damage.

Although all statins share a core structure and inhibit HMG-CoA reductase as their primary mechanism of action, they differ significantly in chemical composition and pharmacokinetics (Sirtori, 2014). Lovastatin, pravastatin, and simvastatin are naturally derived from fungi, while atorvastatin, cerivastatin, fluvastatin, pitavastatin, and rosuvastatin are fully synthetic (ENDO, 2004). Simvastatin and lovastatin are lactone prodrugs that require enzymatic activation to exert their effects. In contrast, atorvastatin, fluvastatin, pravastatin, and rosuvastatin are administered in their active hydroxyacid form, bypassing the need for metabolic conversion (Sirtori, 2014; Hirota et al., 2020; Zheng et al., 2024). Differences in lipophilicity further distinguish these drugs. Atorvastatin, fluvastatin, simvastatin, and lovastatin are lipophilic and can passively diffuse across cell membranes, promoting wider tissue distribution beyond the liver. Conversely, pravastatin and rosuvastatin are hydrophilic due to polar functional groups—a hydroxyl in pravastatin and a methane sulfonamide in rosuvastatin (Schachter, 2005). These chemical differences may explain, in part, the variations in kinase expression and phosphorylation patterns among the statins observed in this study.

Although a previous study reported that simvastatin could directly inhibit EGFR, ERBB2, MET, and SRC (Li et al., 2020), we were unable to replicate these findings using simvastatin, atorvastatin, or cerivastatin using large-scale screening assays. Although differences in methodology, sensitivity or specificity may account for the discrepancy, none of the platforms we employed demonstrated direct inhibition of these kinases or many others by statins. The earlier study used a radioactive kinase assay, whereas we utilized well-established commercial platforms based on TR-FRET, site-directed competition binding assays, and fluorescence-based detection of proteolytic susceptibility. Additional factors that might contribute to the differing results include compound purity, variations in protein constructs, or differences in experimental context.

In the specific context of colorectal cancer, we observed reduced phosphorylation of EGFR and MET following lovastatin treatment, but no significant changes in SRC phosphorylation. These findings suggest that the effects on EGFR and MET are likely indirect and not due to direct kinase inhibition by statins. Interestingly, we did identify direct inhibition of two kinases: CAMK1G and TSSK1B, with IC_50_ values of 8.9 µM and 3.3 µM, respectively. In this sense, further research is needed to determine whether these inhibitory effects occur *in cellulo* and *in vivo* and to elucidate their potential biological significance. CAMK1G and TSSK1B are members of the calcium/calmodulin-dependent protein kinase (CaMK) family (Swulius and Waxham, 2008). CAMK1G has been shown to phosphorylate the transcription factor CREB1, which plays a key role in promoting cancer development and progression by enhancing cellular plasticity and driving metabolic reprogramming (Watson et al., 2021; Ma et al., 2024). In contrast, TSSK1B is involved in the negative regulation of YAP, a central effector of the Hippo signaling pathway, thereby contributing to the suppression of cellular proliferation and oncogenic transformation (Kim et al., 2024).

The reduction of PI3K phosphorylation induced by statins has also been observed in other types of cancer, such as breast and prostate cancer (Park et al., 2013; Beckwitt et al., 2018). In particular, this effect appears to be specific to lipophilic statins such as atorvastatin, simvastatin and lovastatin (Beckwitt et al., 2018), and is not observed with hydrophilic statins such as pravastatin and rosuvastatin (Shiota et al., 2012; Beckwitt et al., 2018). One possible explanation is that lipophilic statins more readily cross the cancer cell membrane, allowing them to exert intracellular effects more efficiently.

Another potential mechanism involves the disruption of lipid rafts due to the cholesterol-lowering effects of statins (Zaborowska et al., 2023). Since PI3K localizes to the cell membrane and participates in signaling pathways that depend on membrane organization and trafficking, disruption of these domains could impair its phosphorylation and downstream signaling. Furthermore, statins have been shown to upregulate PTEN activity, which in turn negatively regulates the PI3K/AKT/mTOR signaling pathway (Wang et al., 2016; Ouahoud et al., 2021). Statins also interfere with protein prenylation, a post-translational modification essential for the proper localization and function of small GTPases such as RAS (Baranyi et al., 2020). By inhibiting prenylation, statins can suppress downstream RAS-mediated signaling pathways, including both the PI3K/AKT/mTOR and MAPK/ERK cascades (Hubbard et al., 2014; Asati et al., 2016). These mechanisms may help explain why supplementation with mevalonate can reverse the inhibitory effects of atorvastatin on PI3K phosphorylation.

HCT116 is a cell line with a KRAS mutation (G13D) that results in constitutive activation and is associated with poor clinical prognosis, whereas CaCo2 cells have wild-type RAS and are linked to an average clinical outcome. It is possible that the presence of constitutively active RAS in HCT116 cells leads to a smaller reduction in PI3K phosphorylation following statin treatment compared to CaCo2 cells. Additionally, upon mevalonate supplementation, HCT116 cells exhibit an almost complete recovery of PI3K phosphorylation, a response not observed in CaCo2 cells. In this sense, the difference between these two cell lines may lie in the RAS mutation and its impact on downstream signaling pathways.

Statins have demonstrated synergistic effects with various chemotherapeutic agents across multiple cancer types. These include drugs such as 5-fluorouracil (5-FU) (Zabielska et al., 2025), doxorubicin (Środa-Pomianek et al., 2019), cyclophosphamide (Cash et al., 2023) and pentoxifylline (Etemadi et al., 2022). Notably, this synergy also extends to tyrosine kinase inhibitors (TKIs), including sorafenib (Cheng et al., 2017), vemurafenib (Theodosakis et al., 2019), and gefitinib (Chen et al., 2013). These observations are in line with the findings of our study, supporting the potential of statins as effective adjuvants that enhance the efficacy of conventional anticancer therapies.

In conclusion, these studies shed new light on potentially important mechanisms of statin signaling and kinases. The pleiotropic effects of statins may involve transcriptional regulation, posttranslational modifications such as phosphorylation, and direct interactions with kinases, other cellular targets, and the cell membrane. Importantly, these mechanisms can vary from statin to statin and are influenced by their simultaneous interactions with multiple kinases. This complexity may help explain why different statins exhibit variable efficacy and tolerability in different individuals, and why switching from one statin to another can sometimes reduce side effects or potentiate hypolipidemic effects.

## Data Availability

The original contributions presented in the study are included in the article/Supplementary Material, further inquiries can be directed to the corresponding authors.

## References

[B1] Ahn J. Y. Schwarz J. K. Piwnica-Worms H. Canman C. E. (2000). Threonine 68 phosphorylation by ataxia telangiectasia mutated is required for efficient activation of Chk2 in response to ionizing radiation. Cancer Res. 60, 5934–5936. Available online at: http://www.ncbi.nlm.nih.gov/pubmed/11085506. 11085506

[B2] Ai L. Xu A. Xu J. (2020). “Roles of PD-1/PD-L1 pathway: Signaling, cancer, and ©eyond,” in Regulation of cancer immune checkpoints, 33–59. 10.1007/978-981-15-3266-5_3 32185706

[B3] Asati V. Mahapatra D. K. Bharti S. K. (2016). PI3K/Akt/mTOR and Ras/Raf/MEK/ERK signaling pathways inhibitors as anticancer agents: structural and pharmacological perspectives. Eur. J. Med. Chem. 109, 314–341. 10.1016/j.ejmech.2016.01.012 26807863

[B4] Baranyi M. Buday L. Hegedűs B. (2020). K-Ras prenylation as a potential anticancer target. Cancer Metastasis Rev. 39, 1127–1141. 10.1007/s10555-020-09902-w 32524209 PMC7680335

[B5] Beckwitt C. H. Shiraha K. Wells A. (2018). Lipophilic statins limit cancer cell growth and survival, *via* involvement of akt signaling. PLoS One 13, e0197422. 10.1371/journal.pone.0197422 29763460 PMC5953490

[B6] Bolger A. M. Lohse M. Usadel B. (2014). Trimmomatic: a flexible trimmer for illumina sequence data. Bioinformatics 30, 2114–2120. 10.1093/bioinformatics/btu170 24695404 PMC4103590

[B7] Bray F. Laversanne M. Sung H. Ferlay J. Siegel R. L. Soerjomataram I. (2024). Global cancer statistics 2022: GLOBOCAN estimates of incidence and mortality worldwide for 36 cancers in 185 countries. Ca. Cancer J. Clin. 74, 229–263. 10.3322/caac.21834 38572751

[B8] Bredefeld C. L. Choi P. Cullen T. Nicolich-Henkin S. J. Waters L. (2024). Statin use and hyperglycemia: do statins cause diabetes? Curr. Atheroscler. Rep. 27, 18. 10.1007/s11883-024-01266-8 39699704

[B9] Cash T. Jonus H. C. Tsvetkova M. Beumer J. H. Sadanand A. Lee J. Y. (2023). A phase 1 study of simvastatin in combination with topotecan and cyclophosphamide in pediatric patients with relapsed and/or refractory solid and CNS tumors. Pediatr. Blood Cancer 70, e30405. 10.1002/pbc.30405 37158620 PMC11225565

[B10] Chen J. Bi H. Hou J. Zhang X. Zhang C. Yue L. (2013). Atorvastatin overcomes gefitinib resistance in KRAS mutant human non-small cell lung carcinoma cells. Cell Death Dis. 4, e814. 10.1038/cddis.2013.312 24071646 PMC3789171

[B11] Cheng Y. Luo R. Zheng H. Wang B. Liu Y. Liu D. (2017). Synergistic anti-tumor efficacy of sorafenib and fluvastatin in hepatocellular carcinoma. Oncotarget 8, 23265–23276. 10.18632/oncotarget.15575 28423574 PMC5410302

[B12] Cozzone A. J. (1998). Post-translational modification of proteins by reversible phosphorylation in prokaryotes. Biochimie 80, 43–48. 10.1016/S0300-9084(98)80055-2 9587661

[B13] Du Z. Lovly C. M. (2018). Mechanisms of receptor tyrosine kinase activation in cancer. Mol. Cancer 17, 58. 10.1186/s12943-018-0782-4 29455648 PMC5817791

[B14] Endo A. (2004). The origin of the statins. Atheroscler. Suppl. 5, 125–130. 10.1016/j.atherosclerosissup.2004.08.033 15531285

[B15] Etemadi S. Abtahi Froushani S. M. Hashemi Asl S. M. Mahmoudian A. (2022). Combined atorvastatin and pentoxifylline in ameliorating inflammation induced by complete Freund’s adjuvant. Inflammopharmacology 30, 935–944. 10.1007/s10787-022-00957-5 35428948

[B16] Hirota T. Fujita Y. Ieiri I. (2020). An updated review of pharmacokinetic drug interactions and pharmacogenetics of statins. Expert Opin. Drug Metab. Toxicol. 16, 809–822. 10.1080/17425255.2020.1801634 32729746

[B17] Hubbard P. A. Moody C. L. Murali R. (2014). Allosteric modulation of ras and the PI3K/AKT/mTOR pathway: emerging therapeutic opportunities. Front. Physiol. 5, 478. 10.3389/fphys.2014.00478 25566081 PMC4267178

[B18] Jiang W. Hu J.-W. He X.-R. Jin W.-L. He X.-Y. (2021). Statins: a repurposed drug to fight cancer. J. Exp. Clin. Cancer Res. 40, 241. 10.1186/s13046-021-02041-2 34303383 PMC8306262

[B19] Juarez D. Fruman D. A. (2021). Targeting the mevalonate pathway in cancer. Trends Cancer 7, 525–540. 10.1016/j.trecan.2020.11.008 33358111 PMC8137523

[B20] Kanehisa M. Goto S. (2000). KEGG: kyoto encyclopedia of genes and genomes. Nucleic Acids Res. 28, 27–30. 10.1093/nar/28.1.27 10592173 PMC102409

[B21] Kass E. M. Poyurovsky M. V. Zhu Y. Prives C. (2009). Mdm2 and PCAF increase Chk2 ubiquitination and degradation independently of their intrinsic E3 ligase activities. Cell Cycle 8, 430–437. 10.4161/cc.8.3.7624 19176998

[B22] Khan K. Valeri N. Dearman C. Rao S. Watkins D. Starling N. (2019). Targeting EGFR pathway in metastatic colorectal cancer-tumour heterogeniety and convergent evolution. Crit. Rev. Oncol. Hematol. 143, 153–163. 10.1016/j.critrevonc.2019.09.001 31678702

[B23] Kim C.-L. Lim S.-B. B. Choi S.-H. Kim D. H. Sim Y. E. Jo E.-H. (2024). The LKB1–TSSK1B axis controls YAP phosphorylation to regulate the Hippo–YAP pathway. Cell Death Dis. 15, 76. 10.1038/s41419-024-06465-4 38245531 PMC10799855

[B24] Kim D. Paggi J. M. Park C. Bennett C. Salzberg S. L. (2019). Graph-based genome alignment and genotyping with HISAT2 and HISAT-genotype. Nat. Biotechnol. 37, 907–915. 10.1038/s41587-019-0201-4 31375807 PMC7605509

[B25] Lagunas-Rangel F. A. (2025a). Bidirectional relationship between statins and the gut microbiota: Implications for Cardiovascular Health, Diabetes, and Cancer. Xenobiotica 10.1080/00498254.2025.2535445 40684299

[B26] Lagunas-Rangel F. A. (2025b). Cholesterol effects on the tumor immune microenvironment: from fundamental concepts to mechanisms and implications. Front. Oncol. 15, 1579054–18. 10.3389/fonc.2025.1579054 40270603 PMC12014580

[B27] Lagunas‐Rangel F. A. Liepinsh E. Fredriksson R. Alsehli A. M. Williams M. J. Dambrova M. (2024). Off‐target effects of statins: molecular mechanisms, side effects and the emerging role of kinases. Br. J. Pharmacol. 181, 3799–3818. 10.1111/bph.17309 39180421

[B28] Lagunas‐Rangel F. A. Liu W. Schiöth H. B. (2023). Interaction between environmental pollutants and cancer drug efficacy: Bisphenol A, bisphenol A diglycidyl ether and perfluorooctanoic acid reduce vincristine cytotoxicity in acute lymphoblastic leukemia cells. J. Appl. Toxicol. 43, 458–469. 10.1002/jat.4398 36181250

[B29] Levantini E. Maroni G. Del Re M. Tenen D. G. (2022). EGFR signaling pathway as therapeutic target in human cancers. Semin. Cancer Biol. 85, 253–275. 10.1016/j.semcancer.2022.04.002 35427766

[B30] Li Y. Wei X. Wang Q. Li W. Yang T. (2020). Inverse screening of simvastatin kinase targets from glioblastoma druggable kinome. Comput. Biol. Chem. 86, 107243. 10.1016/j.compbiolchem.2020.107243 32172201

[B31] Liao Y. Smyth G. K. Shi W. (2014). featureCounts: an efficient general purpose program for assigning sequence reads to genomic features. Bioinformatics 30, 923–930. 10.1093/bioinformatics/btt656 24227677

[B32] Liu R. Chen Y. Liu G. Li C. Song Y. Cao Z. (2020). PI3K/AKT pathway as a key link modulates the multidrug resistance of cancers. Cell Death Dis. 11, 797. 10.1038/s41419-020-02998-6 32973135 PMC7515865

[B33] Ma Y. Zong H. Pan P. Shang H. Yang X. (2024). The CREB1/WNK1 axis promotes the tumorigenesis of ovarian cancer via regulating HIF-1. Exp. Cell Res. 438, 114006. 10.1016/j.yexcr.2024.114006 38599542

[B34] Mach F. Baigent C. Catapano A. L. Koskinas K. C. Casula M. Badimon L. (2020). 2019 ESC/EAS guidelines for the management of dyslipidaemias: lipid modification to reduce cardiovascular risk. Eur. Heart J. 41, 111–188. 10.1093/eurheartj/ehz455 31504418

[B35] Matusewicz L. Czogalla A. Sikorski A. F. (2020). Attempts to use statins in cancer therapy: an update. Tumor Biol. 42, 1010428320941760. 10.1177/1010428320941760 32662332

[B36] Mollazadeh H. Tavana E. Fanni G. Bo S. Banach M. Pirro M. (2021). Effects of statins on mitochondrial pathways. J. Cachexia. Sarcopenia Muscle 12, 237–251. 10.1002/jcsm.12654 33511728 PMC8061391

[B37] Napolitano S. Martini G. Ciardiello D. Del Tufo S. Martinelli E. Troiani T. (2024). Targeting the EGFR signalling pathway in metastatic colorectal cancer. Lancet Gastroenterol. Hepatol. 9, 664–676. 10.1016/S2468-1253(23)00479-X 38697174

[B38] Norkin M. Ordóñez-Morán P. Huelsken J. (2021). High-content, targeted RNA-seq screening in organoids for drug discovery in colorectal cancer. Cell Rep. 35, 109026. 10.1016/j.celrep.2021.109026 33882314

[B39] Ouahoud S. Jacobs R. J. Peppelenbosch M. P. Fühler G. M. Heijmans J. Diks S. (2021). Kinome-wide analysis of the effect of statins in colorectal cancer. Br. J. Cancer 124, 1978–1987. 10.1038/s41416-021-01318-9 33742146 PMC8184819

[B40] Park Y. H. Jung H. H. Ahn J. S. Im Y.-H. (2013). Statin induces inhibition of triple negative breast cancer (TNBC) cells via PI3K pathway. Biochem. Biophys. Res. Commun. 439, 275–279. 10.1016/j.bbrc.2013.08.043 23973711

[B41] Perelas A. Tsoulkani A. Perrea D. (2010). Effects of lipid-lowering drugs on adiponectin. Curr. Vasc. Pharmacol. 8, 836–848. 10.2174/157016110793563870 20180775

[B42] Robinson M. D. McCarthy D. J. Smyth G. K. (2010). edgeR: a Bioconductor package for differential expression analysis of digital gene expression data. Bioinformatics 26, 139–140. 10.1093/bioinformatics/btp616 19910308 PMC2796818

[B43] Schachter M. (2005). Chemical, pharmacokinetic and pharmacodynamic properties of statins: an update. Fundam. Clin. Pharmacol. 19, 117–125. 10.1111/j.1472-8206.2004.00299.x 15660968

[B44] She J. Tuerhongjiang G. Guo M. Liu J. Hao X. Guo L. (2024). Statins aggravate insulin resistance through reduced blood glucagon-like peptide-1 levels in a microbiota-dependent manner. Cell Metab. 36, 408–421.e5. 10.1016/j.cmet.2023.12.027 38325336

[B45] Shiota M. Hikita Y. Kawamoto Y. Kusakabe H. Tanaka M. Izumi Y. (2012). Pravastatin‐induced proangiogenic effects depend upon extracellular FGF‐2. J. Cell. Mol. Med. 16, 2001–2009. 10.1111/j.1582-4934.2011.01494.x 22117815 PMC3822970

[B46] Silvius J. R. (2003). Role of cholesterol in lipid raft formation: lessons from lipid model systems. Biochim. Biophys. Acta - Biomembr. 1610, 174–183. 10.1016/S0005-2736(03)00016-6 12648772

[B47] Singh P. Saxena R. Srinivas G. Pande G. Chattopadhyay A. (2013). Cholesterol biosynthesis and homeostasis in regulation of the cell cycle. PLoS One 8, e58833. 10.1371/journal.pone.0058833 23554937 PMC3598952

[B48] Sirtori C. R. (2014). The pharmacology of statins. Pharmacol. Res. 88, 3–11. 10.1016/j.phrs.2014.03.002 24657242

[B49] Środa-Pomianek K. Michalak K. Palko-Łabuz A. Uryga A. Świątek P. Majkowski M. (2019). The combined use of phenothiazines and statins strongly affects doxorubicin-resistance, apoptosis, and Cox-2 activity in Colon cancer cells. Int. J. Mol. Sci. 20, 955. 10.3390/ijms20040955 30813251 PMC6412564

[B50] Swulius M. T. Waxham M. N. (2008). Ca2+/Calmodulin-dependent protein kinases. Cell. Mol. Life Sci. 65, 2637–2657. 10.1007/s00018-008-8086-2 18463790 PMC3617042

[B51] Theodosakis N. Langdon C. G. Micevic G. Krykbaeva I. Means R. E. Stern D. F. (2019). Inhibition of isoprenylation synergizes with MAPK blockade to prevent growth in treatment‐resistant melanoma, colorectal, and lung cancer. Pigment. Cell Melanoma Res. 32, 292–302. 10.1111/pcmr.12742 30281931 PMC6590911

[B52] Wang T. Seah S. Loh X. Chan C.-W. Hartman M. Goh B.-C. (2016). Simvastatin-induced breast cancer cell death and deactivation of PI3K/Akt and MAPK/ERK signalling are reversed by metabolic products of the mevalonate pathway. Oncotarget 7, 2532–2544. 10.18632/oncotarget.6304 26565813 PMC4823053

[B53] Watson M. J. Berger P. L. Banerjee K. Frank S. B. Tang L. Ganguly S. S. (2021). Aberrant CREB1 activation in prostate cancer disrupts normal prostate luminal cell differentiation. Oncogene 40, 3260–3272. 10.1038/s41388-021-01772-y 33846571 PMC10760404

[B54] Wingett S. W. Andrews S. (2018). FastQ screen: a tool for multi-genome mapping and quality control. F1000Research 7, 1338. 10.12688/f1000research.15931.2 30254741 PMC6124377

[B55] Xiao M. Xu J. Wang W. Zhang B. Liu J. Li J. (2023). Functional significance of cholesterol metabolism in cancer: from threat to treatment. Exp. Mol. Med. 55, 1982–1995. 10.1038/s12276-023-01079-w 37653037 PMC10545798

[B56] Xie Y.-H. Chen Y.-X. Fang J.-Y. (2020). Comprehensive review of targeted therapy for colorectal cancer. Signal Transduct. Target. Ther. 5, 22. 10.1038/s41392-020-0116-z 32296018 PMC7082344

[B57] Zabielska J. Stelmanska E. Szrok-Jurga S. Kobiela J. Czumaj A. (2025). Lipids metabolism inhibition antiproliferative synergy with 5-Fluorouracil in human colorectal cancer model. Int. J. Mol. Sci. 26, 1186. 10.3390/ijms26031186 39940954 PMC11818398

[B58] Zaborowska M. Broniatowski M. Fontaine P. Bilewicz R. Matyszewska D. (2023). Statin action targets lipid rafts of cell membranes: GIXD/PM-IRRAS investigation of langmuir monolayers. J. Phys. Chem. B 127, 7135–7147. 10.1021/acs.jpcb.3c02574 37551973 PMC10440791

[B59] Zheng E. Madura P. Grandos J. Broncel M. Pawlos A. Woźniak E. (2024). When the same treatment has different response: the role of pharmacogenomics in Statin therapy. Biomed. Pharmacother. 170, 115966. 10.1016/j.biopha.2023.115966 38061135

