# Supplementary material for: Statins regulate kinase signaling by causing changes in phosphorylation, rather than through changes in gene expression or direct inhibition: evidence in colorectal cancer
Source: Front Pharmacol. 2025 Aug 4;16:1653702. doi: 10.3389/fphar.2025.1653702 (PMC12358271; doi:10.3389/fphar.2025.1653702)
Supplement: Supplementary file 1 [file Table1.docx]

Supplementary Tables

**Supplementary Table 1. Results of *in vitro* kinome profiling assay using atorvastatin, simvastatin, and fluvastatin**

|  |  | **% Average inhibition (n=3)** | | |
| --- | --- | --- | --- | --- |
| **Kinase** | **Methodology** | **Atorvastatin (1000 nM)** | **Cerivastatin**  **(1000 nM)** | **Simvastatin (1000 nM)** |
| BRAF V599E | ZLYTE | -13 | -14 | -18 |
| ABL1 | ZLYTE | -7 | -15 | -19 |
| CAMK1 (CaMK1) | Adapta | -15 | -7 | -17 |
| MAPK9 (JNK2) | ZLYTE | -2 | -17 | -13 |
| MAPKAPK2 | ZLYTE | -6 | -12 | -13 |
| PIP5K1C | Adapta | -10 | -3 | -18 |
| RIPK3 | LanthaScreen Binding | -5 | -16 | -9 |
| MAP2K4 (MEK4) | LanthaScreen Binding | -3 | -12 | -13 |
| PIK3CA E545K/PIK3R1 (p110 alpha E545K/p85 alpha) | Adapta | -8 | -11 | -8 |
| MARK4 | ZLYTE | -5 | -8 | -13 |
| GSG2 (Haspin) | Adapta | -16 | -4 | -5 |
| LRRK2 G2019S | Adapta | -6 | -11 | -8 |
| MET (cMet) Y1235D | ZLYTE | -7 | -5 | -12 |
| SRC | ZLYTE | -8 | -3 | -13 |
| LIMK1 | LanthaScreen Binding | -2 | -15 | -6 |
| EEF2K | ZLYTE | -3 | -9 | -10 |
| EPHA8 | ZLYTE | -9 | -4 | -9 |
| MAP3K8 (COT) | ZLYTE | 5 | -15 | -12 |
| PRKACB (PRKAC beta) | LanthaScreen Binding | -10 | -11 | -1 |
| TEK (TIE2) R849W | LanthaScreen Binding | -1 | -12 | -9 |
| TGFBR2 | LanthaScreen Binding | -5 | -15 | -2 |
| BRAF | ZLYTE | -3 | -8 | -10 |
| MAP2K6 (MKK6) S207E T211E | LanthaScreen Binding | -11 | -4 | -6 |
| MAP3K5 (ASK1) | LanthaScreen Binding | 4 | -3 | -20 |
| LATS2 | LanthaScreen Binding | 1 | -16 | -3 |
| MAP2K1 (MEK1) | ZLYTE | 3 | -8 | -13 |
| PIK3CB/PIK3R2 (p110 beta/p85 beta) | Adapta | -11 | -7 | 0 |

| PRKCZ (PKC zeta) | ZLYTE | -2 | -7 | -9 |
| --- | --- | --- | --- | --- |
| ABL1 E255K | ZLYTE | -2 | -9 | -6 |
| FLT3 ITD | LanthaScreen Binding | -9 | -3 | -5 |
| MYO3B (MYO3 beta) | LanthaScreen Binding | -6 | -6 | -4 |
| PIK3C3 (hVPS34) | Adapta | -1 | -2 | -13 |
| PIK3CD/PIK3R1 (p110 delta/p85 alpha) | Adapta | -6 | 0 | -10 |
| SGK (SGK1) | ZLYTE | 0 | -8 | -8 |
| PRKCG (PKC gamma) | ZLYTE | -3 | -4 | -7 |
| RIPK2 | LanthaScreen Binding | -6 | -3 | -5 |
| TESK1 | LanthaScreen Binding | -2 | -12 | 0 |
| FYN | ZLYTE | 5 | -9 | -9 |
| PRKCB1 (PKC beta I) | ZLYTE | -4 | -5 | -4 |
| SPHK1 | Adapta | -3 | 0 | -10 |
| STK38L (NDR2) | LanthaScreen Binding | -8 | 4 | -9 |
| MAP2K5 (MEK5) | LanthaScreen Binding | 4 | -3 | -12 |
| MASTL | LanthaScreen Binding | 1 | -10 | -2 |
| BMPR2 | LanthaScreen Binding | -5 | 2 | -7 |
| CDK16 (PCTK1)/cyclin Y | LanthaScreen Binding | -2 | 2 | -10 |
| GRK4 | ZLYTE | -5 | -4 | -1 |
| GRK7 | ZLYTE | -4 | -4 | -2 |
| MATK (HYL) | ZLYTE | 2 | -4 | -8 |
| NEK9 | ZLYTE | -3 | -4 | -3 |
| NLK | LanthaScreen Binding | -1 | -6 | -3 |
| RET A883F | ZLYTE | -1 | -7 | -2 |
| TESK2 | LanthaScreen Binding | -6 | 0 | -4 |
| CDK17/cyclin Y | ZLYTE | -1 | -3 | -5 |
| EIF2AK2 (PKR) | LanthaScreen Binding | 6 | -4 | -11 |
| EPHA6 | LanthaScreen Binding | -3 | -4 | -2 |
| NEK2 | ZLYTE | 0 | -4 | -5 |
| PRKCN (PKD3) | ZLYTE | -3 | 0 | -6 |
| SRMS (Srm) | ZLYTE | 0 | -5 | -4 |
| ACVR2A | LanthaScreen Binding | 0 | -2 | -6 |
| FGFR4 | ZLYTE | 1 | -6 | -3 |

| AURKA (Aurora A) | ZLYTE | -1 | -3 | -3 |
| --- | --- | --- | --- | --- |
| CAMKK2 (CaMKK beta) | LanthaScreen Binding | -2 | -7 | 2 |
| CLK4 | LanthaScreen Binding | 0 | 2 | -9 |
| EGFR (ErbB1) C797S | ZLYTE | -2 | 1 | -6 |
| MINK1 | ZLYTE | 2 | -6 | -3 |
| MYLK (MLCK) | LanthaScreen Binding | -3 | -3 | -1 |
| NIM1K | ZLYTE | 0 | -3 | -4 |
| SPHK2 | Adapta | -12 | 5 | 0 |
| TNK1 | ZLYTE | -2 | -3 | -2 |
| AMPK (A1/B2/G3) | ZLYTE | -3 | -4 | 1 |
| BLK | ZLYTE | 4 | -3 | -7 |
| CDK2/cyclin A | ZLYTE | -2 | -1 | -3 |
| CDK9/cyclin K | LanthaScreen Binding | -1 | -1 | -4 |
| DMPK | LanthaScreen Binding | -1 | -3 | -2 |
| DYRK1A | ZLYTE | -3 | -2 | -1 |
| EPHA1 | ZLYTE | 2 | -8 | 0 |
| IRAK4 | ZLYTE | -1 | -3 | -2 |
| MAP2K6 (MKK6) | ZLYTE | 2 | -7 | -1 |
| NEK8 | LanthaScreen Binding | 1 | -7 | 0 |
| STK25 (YSK1) | ZLYTE | 1 | 0 | -7 |
| AKT1 (PKB alpha) | ZLYTE | 0 | -2 | -3 |
| BMPR1A (ALK3) | LanthaScreen Binding | 1 | -4 | -2 |
| CAMKK1 (CAMKKA) | LanthaScreen Binding | 2 | -3 | -4 |
| DCAMKL1 (DCLK1) | ZLYTE | 2 | -1 | -6 |
| SLK | LanthaScreen Binding | -3 | -1 | -1 |
| STK38 (NDR) | LanthaScreen Binding | -1 | -7 | 3 |
| ACVR1B (ALK4) | ZLYTE | 3 | -3 | -4 |
| AMPK A1/B1/G1 | ZLYTE | 2 | -3 | -3 |
| EPHA4 | ZLYTE | -4 | -1 | 1 |
| FLT3 D835Y | ZLYTE | 2 | 0 | -6 |
| IRAK3 | LanthaScreen Binding | -11 | 2 | 5 |
| MAP3K14 (NIK) | LanthaScreen Binding | 3 | -1 | -6 |
| MAP3K2 (MEKK2) | LanthaScreen Binding | -4 | -5 | 5 |

| MAPK3 (ERK1) | ZLYTE | 0 | -2 | -2 |
| --- | --- | --- | --- | --- |
| PKN1 (PRK1) | ZLYTE | -2 | 1 | -3 |
| SRPK2 | ZLYTE | 1 | -3 | -2 |
| TAOK2 (TAO1) | ZLYTE | 1 | -3 | -2 |
| CSNK1G2 (CK1 gamma 2) | ZLYTE | 0 | -3 | 0 |
| DDR2 | LanthaScreen Binding | -2 | 0 | -1 |
| JAK2 | ZLYTE | 1 | -2 | -2 |
| NUAK1 (ARK5) | Adapta | -5 | 2 | 0 |
| PI4KA (PI4K alpha) | Adapta | 5 | -5 | -3 |
| PIK3C2A (PI3K | C2 alpha) | -4 | 0 | 1 |
| ROCK1 | ZLYTE | -3 | 1 | -1 |
| ROCK2 | ZLYTE | 1 | 0 | -4 |
| TLK2 | LanthaScreen Binding | -2 | -12 | 11 |
| ADRBK1 (GRK2) | ZLYTE | 2 | -2 | -2 |
| DAPK1 | Adapta | -1 | -2 | 1 |
| DDR1 | LanthaScreen Binding | 0 | -2 | 0 |
| EGFR (ErbB1) | ZLYTE | -1 | -1 | 0 |
| FGR | ZLYTE | -2 | 1 | -1 |
| MAP2K2 (MEK2) | ZLYTE | 3 | 1 | -6 |
| MAP3K10 (MLK2) | LanthaScreen Binding | -7 | 0 | 5 |
| MET (cMet) | ZLYTE | 2 | 5 | -9 |
| MKNK2 (MNK2) | LanthaScreen Binding | -1 | 2 | -3 |
| PASK | ZLYTE | 0 | -1 | -1 |
| PIK3CA/PIK3R1 (p110 alpha/p85 alpha) | Adapta | -3 | 0 | 1 |
| PIK3CG (p110 gamma) | Adapta | -5 | 3 | 0 |
| PRKD2 (PKD2) | ZLYTE | 10 | -4 | -8 |
| ACVR1 (ALK2) | LanthaScreen Binding | 1 | -2 | 0 |
| ADRBK2 (GRK3) | ZLYTE | -1 | -1 | 1 |
| AMPK A2/B1/G1 | ZLYTE | 5 | -3 | -3 |
| CDK2/cyclin A | ZLYTE | 2 | -2 | -1 |
| CDK2/cyclin E1 | LanthaScreen Binding | -7 | -2 | 8 |
| CSNK1G3 (CK1 gamma 3) | ZLYTE | -1 | 0 | 0 |
| EPHA2 | ZLYTE | 2 | -1 | -2 |

| EPHB1 | ZLYTE | 0 | -1 | 0 |
| --- | --- | --- | --- | --- |
| MLK4 | LanthaScreen Binding | -2 | 0 | 1 |
| PAK4 | ZLYTE | -1 | 4 | -4 |
| PHKG1 | ZLYTE | 1 | 1 | -3 |
| PIK3CA/PIK3R3 (p110 alpha/p55 gamma) | Adapta | 4 | -1 | -4 |
| PRKG2 (PKG2) | ZLYTE | 1 | 1 | -3 |
| SGKL (SGK3) | ZLYTE | 0 | -5 | 4 |
| STK23 (MSSK1) | ZLYTE | 0 | 0 | -1 |
| TNK2 (ACK) | LanthaScreen Binding | -3 | 1 | 1 |
| WNK3 | LanthaScreen Binding | 4 | -4 | -1 |
| ALK C1156Y | LanthaScreen Binding | 2 | 6 | -8 |
| AMPK (A2/B2/G3) | ZLYTE | 2 | 0 | -2 |
| CDC42 BPG (MRCKG) | ZLYTE | 0 | -1 | 1 |
| CDK4/cyclin D3 | Adapta | 0 | 1 | -1 |
| EPHB3 | ZLYTE | 0 | 0 | 0 |
| FGFR3 K650E | ZLYTE | 1 | 0 | -1 |
| FLT3 | ZLYTE | 1 | 0 | -1 |
| FRAP1 (mTOR) | ZLYTE | 2 | -3 | 1 |
| GRK5 | ZLYTE | -1 | -1 | 2 |
| INSR | ZLYTE | 2 | 0 | -2 |
| JAK3 | ZLYTE | 1 | -3 | 2 |
| MAP3K9 (MLK1) | ZLYTE | 0 | 1 | -1 |
| MAP4K2 (GCK) | ZLYTE | 1 | 2 | -3 |
| MST4 | ZLYTE | 1 | 0 | -1 |
| PIK3C2G (PI3K | C2 gamma) | -1 | 1 | 0 |
| RPS6KA2 (RSK3) | ZLYTE | 6 | -5 | -1 |
| STK17B (DRAK2) | LanthaScreen Binding | -1 | 1 | 0 |
| STK24 (MST3) | ZLYTE | 0 | 1 | -1 |
| STK32B (YANK2) | LanthaScreen Binding | -19 | 1 | 18 |
| DAPK3 (ZIPK) | ZLYTE | 4 | 1 | -4 |
| HIPK2 | ZLYTE | 0 | 1 | 0 |
| INSRR (IRR) | ZLYTE | 1 | 1 | -1 |
| KDR (VEGFR2) | ZLYTE | 4 | -2 | -1 |

| LYN A | ZLYTE | 6 | -1 | -4 |
| --- | --- | --- | --- | --- |
| MAP4K5 (KHS1) | ZLYTE | 3 | 0 | -2 |
| MERTK (cMER) | ZLYTE | 5 | 1 | -5 |
| MKNK1 (MNK1) | ZLYTE | 3 | -1 | -1 |
| RAF1 (cRAF) Y340D Y341D | LanthaScreen Binding | 0 | 4 | -3 |
| TEC | LanthaScreen Binding | -1 | 0 | 2 |
| CDK18/cyclin Y | ZLYTE | -1 | 1 | 2 |
| CDK9/cyclin T1 | Adapta | -1 | -1 | 4 |
| CSK | ZLYTE | 1 | 0 | 1 |
| DYRK3 | ZLYTE | -2 | 1 | 3 |
| ERBB2 (HER2) | ZLYTE | 2 | 1 | -1 |
| ERBB4 (HER4) | ZLYTE | 2 | 1 | -1 |
| FLT4 (VEGFR3) | ZLYTE | 1 | 1 | 0 |
| HIPK3 (YAK1) | ZLYTE | 2 | 1 | -1 |
| MAP3K19 (YSK4) | ZLYTE | 2 | 2 | -2 |
| MAPK11 (p38 beta) | ZLYTE | 2 | -1 | 1 |
| NEK4 | ZLYTE | 1 | 1 | 0 |
| PAK2 (PAK65) | ZLYTE | 1 | 1 | 0 |
| PDGFRB (PDGFR beta) | ZLYTE | 0 | 1 | 1 |
| PI4K2B (PI4K2 beta) | Adapta | 4 | -1 | -1 |
| PIM2 | ZLYTE | 0 | 2 | 0 |
| STK3 (MST2) | ZLYTE | -1 | -1 | 4 |
| TXK | ZLYTE | 4 | 0 | -2 |
| AURKB (Aurora B) | ZLYTE | 2 | 0 | 1 |
| CDC42 BPA (MRCKA) | ZLYTE | 2 | -1 | 2 |
| CDK1/cyclin B | ZLYTE | 1 | 4 | -2 |
| CDK2/cyclin O | LanthaScreen Binding | 6 | 0 | -3 |
| CDK6/cyclin D1 | Adapta | -1 | 4 | 0 |
| CSNK1G1 (CK1 gamma 1) | ZLYTE | 0 | 0 | 3 |
| DYRK1B | ZLYTE | 3 | 0 | 0 |
| EPHA5 | ZLYTE | 0 | 2 | 1 |
| HIPK1 (Myak) | ZLYTE | 1 | 1 | 1 |
| HUNK | LanthaScreen Binding | 0 | -3 | 6 |

| ITK | ZLYTE | -1 | 4 | 0 |
| --- | --- | --- | --- | --- |
| JAK1 | ZLYTE | 2 | 2 | -1 |
| JAK2 JH1 JH2 | ZLYTE | 2 | 1 | 0 |
| MAP3K11 (MLK3) | LanthaScreen Binding | 4 | -4 | 3 |
| MAPK12 (p38 gamma) | ZLYTE | 4 | -1 | 0 |
| MAPKAPK5 (PRAK) | ZLYTE | 4 | 0 | -1 |
| MELK | ZLYTE | 4 | -2 | 1 |
| NEK1 | ZLYTE | 2 | 1 | 0 |
| PHKG2 | ZLYTE | 2 | 1 | 0 |
| PRKACA (PKA) | ZLYTE | 1 | 1 | 1 |
| PRKCH (PKC eta) | ZLYTE | 5 | 3 | -5 |
| STK16 (PKL12) | LanthaScreen Binding | 5 | -1 | -1 |
| AKT2 (PKB beta) | ZLYTE | 2 | 2 | 0 |
| AMPK (A1/B2/G2) | ZLYTE | 0 | 2 | 2 |
| BRSK2 | LanthaScreen Binding | 3 | -1 | 2 |
| CDC42 BPB (MRCKB) | ZLYTE | 1 | 0 | 3 |
| CDK7/cyclin H/MNAT1 | Adapta | -4 | 7 | 1 |
| EPHB2 | ZLYTE | 2 | 2 | 0 |
| FGFR3 | ZLYTE | 2 | 2 | 0 |
| GRK6 | ZLYTE | 0 | 4 | 0 |
| KSR2 | ZLYTE | 2 | 0 | 2 |
| LRRK2 | Adapta | 1 | -1 | 4 |
| LTK (TYK1) | ZLYTE | -2 | 7 | -1 |
| MLCK (MLCK2) | LanthaScreen Binding | 1 | 1 | 2 |
| PLK2 | ZLYTE | 1 | 1 | 2 |
| ROS1 | ZLYTE | 5 | -2 | 1 |
| AAK1 | LanthaScreen Binding | 4 | -4 | 5 |
| ABL1 T315I | ZLYTE | -2 | -1 | 8 |
| AKT3 (PKB gamma) | ZLYTE | 3 | 2 | 0 |
| AURKC (Aurora C) | ZLYTE | 4 | 0 | 1 |
| BMX | ZLYTE | 4 | -1 | 2 |
| CLK1 | ZLYTE | 3 | 1 | 1 |
| FER | ZLYTE | 2 | 4 | -1 |

| LIMK2 | LanthaScreen Binding | 0 | 4 | 1 |
| --- | --- | --- | --- | --- |
| MAPKAPK3 | ZLYTE | 0 | -1 | 6 |
| MARK3 | ZLYTE | 8 | 1 | -4 |
| PAK7 (KIAA1264) | ZLYTE | 0 | 2 | 3 |
| PIM1 | ZLYTE | 1 | -2 | 6 |
| RPS6KA4 (MSK2) | ZLYTE | 1 | 2 | 2 |
| TAOK3 (JIK) | LanthaScreen Binding | 4 | -3 | 4 |
| ULK2 | LanthaScreen Binding | -1 | 1 | 5 |
| AMPK (A1/B2/G1) | LanthaScreen Binding | 4 | -1 | 3 |
| CDK2/cyclin A1 | LanthaScreen Binding | 18 | -17 | 5 |
| CLK3 | ZLYTE | 1 | 3 | 2 |
| EPHA5 | ZLYTE | 2 | 2 | 2 |
| FGFR2 | ZLYTE | 1 | 1 | 4 |
| PRKCI (PKC iota) | ZLYTE | 1 | 3 | 2 |
| PRKG1 | ZLYTE | 5 | 1 | 0 |
| RPS6KA6 (RSK4) | ZLYTE | 0 | 1 | 5 |
| SIK1 | LanthaScreen Binding | 1 | 6 | -1 |
| TBK1 | ZLYTE | 5 | 2 | -1 |
| ZAP70 | ZLYTE | 5 | 1 | 0 |
| CAMK2A (CaMKII alpha) | ZLYTE | 2 | 2 | 3 |
| FRK (PTK5) | ZLYTE | 5 | 1 | 1 |
| GAK | LanthaScreen Binding | 1 | 1 | 5 |
| HIPK4 | ZLYTE | 3 | 3 | 1 |
| IKBKB (IKK beta) | ZLYTE | 3 | 0 | 4 |
| MAPK14 (p38 alpha) | ZLYTE | 8 | -2 | 1 |
| MYLK4 | LanthaScreen Binding | 0 | 8 | -1 |
| NTRK2 (TRKB) | ZLYTE | 3 | 4 | 0 |
| ABL2 (Arg) | ZLYTE | -6 | 11 | 3 |
| AXL | ZLYTE | 1 | 2 | 5 |
| CDK5/p35 | ZLYTE | 1 | 4 | 3 |
| CSNK1D (CK1 delta) | ZLYTE | 6 | 4 | -2 |
| DCAMKL2 (DCK2) | ZLYTE | 4 | 4 | 0 |
| DNA | PK | 9 | -1 | 0 |

| FGFR1 | ZLYTE | 3 | 4 | 1 |
| --- | --- | --- | --- | --- |
| FLT1 (VEGFR1) | ZLYTE | 0 | 1 | 7 |
| GSK3A (GSK3 alpha) | ZLYTE | 1 | 4 | 3 |
| MAP4K1 (HPK1) | LanthaScreen Binding | 1 | 3 | 4 |
| MAPK15 (ERK7) | LanthaScreen Binding | 4 | 2 | 2 |
| MARK2 | ZLYTE | 6 | 3 | -1 |
| NUAK2 | LanthaScreen Binding | 6 | 1 | 1 |
| PDGFRA (PDGFR alpha) | ZLYTE | 1 | 4 | 3 |
| PRKACG (PRKAC gamma) | LanthaScreen Binding | -3 | 1 | 10 |
| PRKCA (PKC alpha) | ZLYTE | 2 | 5 | 1 |
| PRKCB2 (PKC beta II) | ZLYTE | 4 | 4 | 0 |
| PTK2B (FAK2) | ZLYTE | 8 | 1 | -1 |
| RET | ZLYTE | 2 | 3 | 3 |
| RPS6KA1 (RSK1) | ZLYTE | 2 | 2 | 4 |
| STK4 (MST1) | ZLYTE | 0 | 6 | 2 |
| AMPK (A2/B2/G2) | LanthaScreen Binding | 6 | -4 | 7 |
| CHEK2 (CHK2) | ZLYTE | 6 | 0 | 3 |
| EPHA3 | LanthaScreen Binding | 5 | 2 | 2 |
| MAP3K7/MAP3K7IP1 (TAK1 | TAB1) | -2 | 9 | 2 |
| MAP4K3 (GLK) | LanthaScreen Binding | 2 | 7 | 0 |
| MAPK10 (JNK3) | ZLYTE | 1 | 3 | 5 |
| MST1R (RON) | ZLYTE | 0 | 5 | 4 |
| PIM3 | ZLYTE | 4 | 3 | 2 |
| PIP5K1A | Adapta | 8 | 6 | -5 |
| PIP5K1B | Adapta | 14 | -6 | 1 |
| PRKCB2 (PKC beta II) | ZLYTE | 7 | 3 | -1 |
| PTK6 (Brk) | ZLYTE | 4 | 3 | 2 |
| SIK3 | LanthaScreen Binding | 5 | -5 | 9 |
| SRC N1 | ZLYTE | 3 | 2 | 4 |
| ULK1 | LanthaScreen Binding | 6 | 2 | 1 |
| AMPK (A1/B1/G3) | LanthaScreen Binding | 5 | 5 | 0 |
| AMPK (A2/B1/G3) | ZLYTE | 2 | 5 | 3 |
| CDC7/DBF4 | LanthaScreen Binding | -1 | 7 | 4 |

| CSNK1A1L | ZLYTE | 3 | 5 | 2 |
| --- | --- | --- | --- | --- |
| CSNK1E (CK1 epsilon) | ZLYTE | 0 | 4 | 6 |
| CSNK2A2 (CK2 alpha 2) | ZLYTE | 3 | 6 | 1 |
| GSK3B (GSK3 beta) | ZLYTE | 0 | 3 | 7 |
| ICK | LanthaScreen Binding | 1 | 6 | 3 |
| MAPK1 (ERK2) | ZLYTE | 3 | 4 | 3 |
| SRPK1 | ZLYTE | 0 | 2 | 8 |
| TYK2 | ZLYTE | 1 | 6 | 3 |
| CSF1R (FMS) | ZLYTE | 4 | 5 | 2 |
| IKBKE (IKK epsilon) | ZLYTE | 5 | 2 | 4 |
| MAP2K1 (MEK1) S218D S222D | LanthaScreen Binding | 4 | 1 | 6 |
| MAP3K3 (MEKK3) | LanthaScreen Binding | 4 | 1 | 6 |
| PDK1 | ZLYTE | 8 | 1 | 2 |
| PKN2 (PRK2) | LanthaScreen Binding | -2 | 5 | 8 |
| RPS6KA3 (RSK2) | ZLYTE | 8 | 3 | 0 |
| SBK1 | ZLYTE | 5 | 3 | 3 |
| TEK (Tie2) | ZLYTE | 6 | 3 | 2 |
| CAMK4 (CaMKIV) | ZLYTE | 10 | 0 | 2 |
| CHEK1 (CHK1) | ZLYTE | 9 | 0 | 3 |
| CLK2 | ZLYTE | 3 | 4 | 5 |
| MAPK8 (JNK1) | ZLYTE | 9 | 0 | 3 |
| PRKCE (PKC epsilon) | ZLYTE | 2 | 6 | 4 |
| PRKX | ZLYTE | 6 | 5 | 1 |
| TTK | LanthaScreen Binding | 8 | 2 | 2 |
| YES1 | ZLYTE | 4 | 5 | 3 |
| CDK8/cyclin C | LanthaScreen Binding | 3 | 3 | 7 |
| PIK3C2B (PI3K | C2 beta) | 6 | 2 | 5 |
| WNK2 | LanthaScreen Binding | 9 | 2 | 2 |
| AMPK (A1/B1/G2) | LanthaScreen Binding | 11 | -5 | 8 |
| CAMK2D (CaMKII delta) | ZLYTE | 11 | 2 | 1 |
| MYLK2 (skMLCK) | ZLYTE | 3 | 4 | 7 |
| PAK3 | ZLYTE | 3 | 7 | 4 |
| PIP4K2A | Adapta | 8 | 4 | 2 |

| TBK1 | ZLYTE | 6 | 3 | 5 |
| --- | --- | --- | --- | --- |
| PIP4K2A |  | 8 | 4 | 2 |
| ACVR2B | LanthaScreen Binding | 8 | -4 | 11 |
| EEF2K | ZLYTE | 3 | 5 | 7 |
| FGFR1 V561M | LanthaScreen Binding | 8 | 2 | 5 |
| KIT D816H | LanthaScreen Binding | 6 | 6 | 3 |
| MAPK13 (p38 delta) | ZLYTE | 7 | 4 | 4 |
| PLK1 | ZLYTE | 4 | 7 | 4 |
| STK22B (TSSK2) | ZLYTE | 5 | 6 | 4 |
| STK22D (TSSK1) | ZLYTE | 3 | 7 | 5 |
| CAMK1D (CaMKI delta) | ZLYTE | 5 | 6 | 5 |
| MARK1 (MARK) | ZLYTE | 5 | 5 | 6 |
| PRKCB1 (PKC beta I) | ZLYTE | 7 | 9 | 0 |
| PRKCD (PKC delta) | ZLYTE | 6 | 9 | 1 |
| STK33 | LanthaScreen Binding | 3 | 2 | 11 |
| TAOK1 | LanthaScreen Binding | 3 | 5 | 8 |
| BMPR1B (ALK6) | LanthaScreen Binding | 4 | 6 | 7 |
| CDKL5 | ZLYTE | 8 | 7 | 2 |
| DYRK2 | LanthaScreen Binding | 8 | 8 | 1 |
| EPHB4 | ZLYTE | 10 | 2 | 5 |
| IRAK1 | Adapta | 6 | 5 | 6 |
| MAP4K4 (HGK) | ZLYTE | 6 | 4 | 7 |
| MUSK | ZLYTE | 3 | 7 | 7 |
| SGK2 | ZLYTE | 4 | 7 | 6 |
| CDK11/cyclin C | LanthaScreen Binding | -2 | 2 | 18 |
| DYRK4 | ZLYTE | 3 | 8 | 7 |
| JAK2 JH1 JH2 V617F | ZLYTE | 5 | 7 | 6 |
| NTRK3 (TRKC) | ZLYTE | 10 | 2 | 6 |
| STK17A (DRAK1) | LanthaScreen Binding | 9 | 9 | 0 |
| TLK1 | LanthaScreen Binding | 0 | 4 | 14 |
| BTK | ZLYTE | 9 | 6 | 4 |
| CAMK2B (CaMKII beta) | ZLYTE | 10 | 3 | 6 |
| CSNK2A1 (CK2 alpha 1) | ZLYTE | 4 | 9 | 6 |

| EPHA7 | LanthaScreen Binding | 11 | 5 | 3 |
| --- | --- | --- | --- | --- |
| HCK | ZLYTE | 4 | 11 | 4 |
| MAP4K5 (KHS1) | ZLYTE | 4 | 8 | 7 |
| PI4KB (PI4K beta) | Adapta | 8 | 11 | 0 |
| RPS6KA5 (MSK1) | ZLYTE | 3 | 8 | 8 |
| SNF1LK2 | ZLYTE | -1 | 7 | 13 |
| LCK | ZLYTE | 1 | 8 | 11 |
| PRKD1 (PKC mu) | ZLYTE | 6 | 9 | 5 |
| TYRO3 (RSE) | ZLYTE | 6 | 10 | 4 |
| AMPK (A2/B1/G2) | ZLYTE | 9 | 4 | 8 |
| BRSK1 (SAD1) | ZLYTE | -1 | 13 | 9 |
| CHUK (IKK alpha) | Adapta | 4 | 12 | 5 |
| NEK6 | ZLYTE | 6 | 8 | 7 |
| PTK2 (FAK) | ZLYTE | 2 | 9 | 10 |
| ALK | ZLYTE | 5 | 9 | 8 |
| GRK1 | LanthaScreen Binding | 4 | 4 | 14 |
| PI4K2A (PI4K2 alpha) | Adapta | 6 | 9 | 7 |
| CAMK1G (CAMKI gamma) | ZLYTE | 5 | 8 | 10 |
| CDK3/cyclin E1 | LanthaScreen Binding | 14 | 4 | 5 |
| CAMK2G (CaMKII gamma) | LanthaScreen Binding | -2 | 9 | 17 |
| IGF1R | ZLYTE | 3 | 12 | 9 |
| PAK1 | ZLYTE | 6 | 9 | 9 |
| STK32C (YANK3) | LanthaScreen Binding | 14 | 5 | 5 |
| CASK | LanthaScreen Binding | 8 | 11 | 6 |
| FES (FPS) | ZLYTE | 8 | 11 | 6 |
| PAK6 | ZLYTE | -1 | 16 | 10 |
| VRK2 | LanthaScreen Binding | -6 | 19 | 12 |
| PKMYT1 | LanthaScreen Binding | 6 | 14 | 7 |
| CDK13/cyclin K | LanthaScreen Binding | 8 | 14 | 6 |
| NTRK1 (TRKA) | ZLYTE | 15 | -4 | 17 |
| LYN B | ZLYTE | 6 | 13 | 10 |
| KIT | ZLYTE | -1 | 17 | 14 |
| WEE1 | LanthaScreen Binding | 11 | 14 | 5 |

| MYO3A (MYO3 alpha) | LanthaScreen Binding | 17 | -2 | 16 |
| --- | --- | --- | --- | --- |
| ULK3 | LanthaScreen Binding | 22 | 12 | -1 |
| PLK4 | LanthaScreen Binding | 1 | 17 | 16 |
| ACVRL1 (ALK1) | LanthaScreen Binding | -2 | 13 | 24 |
| PLK3 | ZLYTE | 15 | 12 | 11 |
| CDK4/cyclin D1 | Adapta | 10 | 16 | 13 |
| SYK | ZLYTE | 14 | 14 | 12 |
| ERN2 | LanthaScreen Binding | 14 | 12 | 17 |
| MAPK7 (ERK5) | ZLYTE | 12 | 10 | 21 |
| WNK1 | LanthaScreen Binding | 15 | 16 | 12 |

# 
